# Understanding progress in software citation: a study of software citation in the CORD-19 corpus

**DOI:** 10.7717/peerj-cs.1022

**Published:** 2022-07-25

**Authors:** Caifan Du, Johanna Cohoon, Patrice Lopez, James Howison

**Affiliations:** 1 The University of Texas at Austin, Austin, TX, United States of America; 2 Science-miner, Naves, France

**Keywords:** Software citation, Science policy, Scholarly communication

## Abstract

In this paper, we investigate progress toward improved software citation by examining current software citation practices. We first introduce our machine learning based data pipeline that extracts software mentions from the CORD-19 corpus, a regularly updated collection of more than 280,000 scholarly articles on COVID-19 and related historical coronaviruses. We then closely examine a stratified sample of extracted software mentions from recent CORD-19 publications to understand the status of software citation. We also searched online for the mentioned software projects and their citation requests. We evaluate both practices of referencing software in publications and making software citable in comparison with earlier findings and recent advocacy recommendations. We found increased mentions of software versions, increased open source practices, and improved software accessibility. Yet, we also found a continuation of high numbers of informal mentions that did not sufficiently credit software authors. Existing software citation requests were diverse but did not match with software citation advocacy recommendations nor were they frequently followed by researchers authoring papers. Finally, we discuss implications for software citation advocacy and standard making efforts seeking to improve the situation. Our results show the diversity of software citation practices and how they differ from advocacy recommendations, provide a baseline for assessing the progress of software citation implementation, and enrich the understanding of existing challenges.

## Introduction

Software is crucial to research, but its visibility in scholarly records is problematic, undermining research policy goals (Mayernik et al., 2017; Howison & Bullard, 2016; Bouquin et al., 2020). These goals include facilitating more verifiable and reproducible research by explicitly referencing software in scholarly communications (Howison & Bullard, 2016), ensuring sufficient credit for research software work within the scientific reputation economy (Bouquin et al., 2020; Howison & Herbsleb, 2011), and tracking the research impact of software for decisions of funding and support (Katz & Smith, 2015; Mayernik et al., 2017; Allen, Teuben & Ryan, 2018). Making software visible in scholarly communication and evaluation can enable these goals. It is also instrumental to incentivize high quality software work that buttresses more robust and useful research. Thus, there is a need to improve the visibility of software in research. Corresponding advocacy efforts have been gaining traction recently, pushing toward machine- and human-actionable software citations in scholarly communications.

A body of research has investigated software visibility in the scholarly record, with results largely confirming the frustration research software practitioners express (*e.g.*, Howison & Bullard, 2016; Pan et al., 2018; Bouquin et al., 2020). For instance, in contrast with the well established practice of citing publications, Howison and Bullard found through their examination of biology articles published between 2000 and 2010 that software citation “practices are varied and appear relatively ad hoc” (Howison & Bullard, 2016). They found 43% of mentions of software were “informal” mentions without bibliographical references, largely noting the software “name-only”; 39% of mentions appeared in bibliographies; 19% of mentions were presented in a “like instrument” manner which gave the software name and its (usually commercial) publisher with an address. Only 28.5% of the mentions were found to provide version information; only 6% of those versions could be found online. Similarly, Pan et al. (2018) traced specific cases of software through 481 papers and found “researchers mention and cite the tools in diverse ways, many of which fall short of a traditional formal citation”. Finally, Bouquin and colleagues studied practices in the Astronomy literature, examining publisher-provided XML for over 76,000 articles published between 1995 and 2018 (Bouquin et al., 2020). Using a list of nine known software packages, they highlighted the “variation” in how these packages were mentioned. They identified diverse “software aliases” in different locations within papers, highlighting false positives due to name ambiguity (*e.g.*, a package named after a planet). They concluded that while software is valued, the inability to “systematically identify software citations” due to their variability can lead “people to doubt the value of citing software” and the authors highlight the importance of advocacy efforts to standardize practices.

These studies have also noted that it is important that software providers indicate how they would like to be credited within papers. Howison and Bullard found only 18% of mentioned packages provided a “request for citation”, but these requests appeared to be effective: 68% of the mentions of those packages followed those requests. Bouquin and colleagues identified “preferred citations” statements made by software providers online for all their studied packages, reporting that these were often requesting multiple preferred citations or sometimes made inconsistent citation requests across different locations.

Advocacy around software citation has called for new (or clarified) practices among software providers, end-user researchers, and publication venue editors. The FORCE11 organization has been one venue for this work through the Software Citation Working Group (and its follow-up initiatives), leading to the publication of clear recommendations (Smith, Katz & Niemeyer, 2016; Katz et al., 2019; Katz et al., 2021; Chue Hong et al., 2019). This advocacy has also connected with similar issues in the sphere of research data, including DataCite (Brase, Lautenschlager & Sens, 2015) and FAIR principles (Hong et al., 2021). Additional work has focused on the accessibility of software metadata, such as through software catalogs (Allen & Schmidt, 2014; Monteil et al., 2020; Muench et al., 2020). Other efforts have encouraged unambiguous and machine-actionable formats of “requests for citation”, for instance, the CITATION.cff file (Druskat et al., 2021).

Finally, discovery techniques also have advanced for improving software visibility. Prototype systems such as Depsy (Piwowar & Priem, 2016), CiteAs (Du et al., 2021b), and the recently added software citation discovery feature on GitHub (GitHub, 2021) increase the chance that research software is identified and cited. Publication mining has also sought to identify software reported in research papers. Krüger & Schindler (2020) reviewed 18 studies that use different techniques to extract software mentions from articles. Machine learning (ML) approaches are the most likely to scale such discoveries. Accordingly, corpora with manual annotation of software mentions have been developed (*e.g.*, Schindler et al., 2021; Du et al., 2021a). Supervised ML-based software extraction over large collections with acceptable computational performance is now possible (Lopez et al., 2021a; Lopez et al., 2021b; Schindler et al., 2022; Wade & Williams, 2021). Community efforts titled “Habeus Corpus” was launched and continue to investigate these emerging collections of extracted software mentions (Habeas Corpus, 2021).

The growing capability to extract software mentions from publications at large scale now allows us to better assess how software is mentioned in scholarly records. Thus, in this study, we identify the baseline, opportunities, and challenges for the ongoing effort of making software visible in scholarship. For this purpose, we created a combined annotation scheme based on empirical descriptions of software citation practices and recommendations from advocacy. We used that scheme to annotate a stratified sample of a recently released collection of software mentions automatically extracted from the CORD-19 set of open access publications (Lopez et al., 2021a). In this way, we systematically document and analyze existing practices for software citation. Our annotation results enable us to understand the current status of software citation implementation, provide a baseline for future assessment, and compare with previous findings and advocacy recommendations. Through this analysis, we identify changes, challenges, and pathways for software citation implementation and advocacy.

## Research Questions

1. How is software mentioned in recently published literature? Have these patterns changed from previous studies? How do current patterns compare to recently published guidelines for software citation?
2. How widespread are requests for citation? What form do they take? How do these requests compare to recently published guidance?

## Data & Methods

To answer these research questions, we first obtained a sample of software mentions extracted from recent literature, leveraging a ML-based software entity recognition pipeline. Then, we assembled an annotation scheme built upon both past research and existing recommendations for software citation. We also annotated relevant practices that enable the citation of software by looking for and examining the online records of mentioned software.

### CORD-19 dataset

We used CORD-19, the COVID-19 Open Research Dataset (Wang et al., 2020), to obtain software mentions in recent publications. The CORD-19 was initially released in March 2020 by the Allen Institute for AI as a large-scale collection of publications and preprints on COVID-19 and past related coronavirus research. The initial release included about 28,000 papers; the dataset has been growing through regular updates. We used the 22 March 2021 release in the CORD-19 release history as our base corpus for software mention extraction, downloaded from https://www.semanticscholar.org/cord19/download. Out of its 490,904 bibliographical entries, this release includes 274,400 publications that can be uniquely identified by a distinct DOI (Digital Object Identifier) and 238,283 publications that can be uniquely identified by a distinct PubMed ID.^1^ 1Note these two sets have large overlaps. We present these numbers because there is no perfect global identifier in this corpus release.For reasons discussed below, we did not use the released extracted article content in JSON format, but harvested the open access PDFs instead using the identifiers of articles in the dataset as our starting point.

The CORD-19 dataset is not noise-free (Kanakia et al., 2020). According to the metadata released along with the dataset, the earliest publication dates back to 1800s. Given our purpose of understanding the very recent software citation practices, we first extracted software mentions from the full corpus, then concentrated on the extracted mentions from articles published since 2016 for detailed analysis, the same year during which the Software Citation Principles (Smith, Katz & Niemeyer, 2016) was published. Metadata in the CORD-19 dataset release indicate that 80% of its contents were published after 2020, and 87% were published after 2016. This is because the contents of CORD-19 primarily focus on COVID-19, which emerged only in 2019. Thus, its large number of publications in recent years provide a rich base for us to investigate recent software citation practices.

### Software mention extraction

We harvested the open access versions of these articles using the article metadata in the CORD-19 release, including both PDF and structured XML formats where available. This choice allows for more complete and reliable full-text extraction of software using our Softcite pipeline. The main components of the Softcite pipeline include three pieces of software: a full-text PDF harvester (Article Dataset Builder, 2021), a machine learning library for extracting structured content from scholarly PDFs (GROBID, 2021), and a software mention recognizer powered by a set of machine learning and deep learning models (Softcite Software Mention Recognizer, 2021).

Using the Softcite pipeline, we harvested more full-text articles than those released in the CORD-19 JSON corpus. Software mentions then were extracted from these reharvested open access publications. The extraction method is described in detail in (Lopez et al., 2021b). In short, the pipeline obtains PDFs, structures them using GROBID (GROBID, 2021; Lopez, 2009), and runs the software mention recognizer (Softcite Software Mention Recognizer, 2021) based on a fine-tuned SciBERT+CRF model (Beltagy, Lo & Cohan, 2019) trained on the softcite-dataset (Du et al., 2021a).

While Wade & Williams (2021) also published software mention extractions from CORD-19 based on the softcite-dataset, our extraction enhances the performance in a number of ways, mainly: (1) a more complete number of full-text articles in PDF were obtained, along with their DOI metadata retrieved from CrossRef API; (2) additional techniques to cope with the extreme label imbalance caused by the sparsity of software mentions in publications; (3) extraction of additional attributes (version, URL, publisher, context of mention); and (4) entity disambiguation, including normalization and using Wikidata to identify false positives (*e.g.*, the mention of algorithms but not a particular software instantiation). Finally, the pipeline also attaches in-text and bibliographic references to software mentions when available; these references are disambiguated against and matched with their registered CrossRef DOI. This final step allows us to more efficiently examine formal citation of software. Overall, the Softcite pipeline demonstrated good performance when recognizing mentions of software, its version, publisher, and/or URL mentioned together in text (average f1-score 79.1), with acceptable computational performance for processing very large collections of literature in PDF (evaluation reported in Lopez et al., 2021b).

The softcite-dataset that underlies our extraction pipeline of software mentions is human-curated annotations of biomedical and economic literature, with the majority of annotated software mentions identified in biomedical research publications. The shared focus on biomedicine therefore makes this training set line up well with the examination of the CORD-19 papers.

### Descriptive statistics of extracted software

We published the full extraction results as the Softcite-CORD-19 dataset under CC-BY−4.0 (Lopez et al., 2021a). In this study, we used published version 0.2.1 (Lopez et al., 2021a), which is based on the CORD-19 dataset release on 22 March 2021. This version contains total 295,609 mentions of software, including their semantic and layout details, bibliographical references linking to the in-text software mentions (N = 55,407), and metadata of all the documents in which software mentions are recognized (N = 76,448; out of total 211,213 re-harvested open access full-text documents).

**Figure 1:**
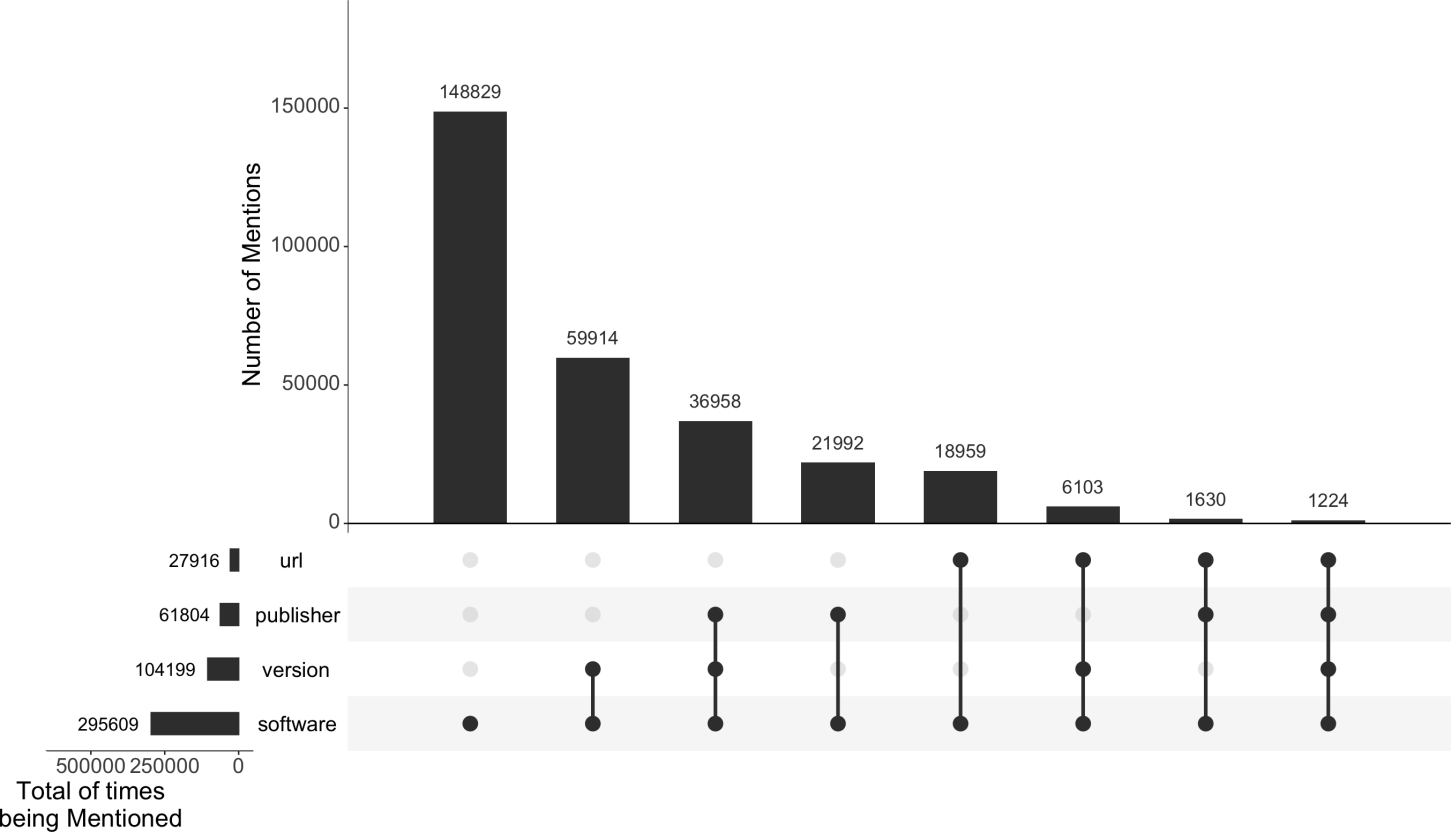
Number of extracted software mentions and their associated details in the Softcite-CORD-19 dataset. The figure was created using UpSetR (Conway, Lex & Gehlenborg, 2017).

Figure 1 shows an overall breakdown of all the 295,609 software mentions in the Softcite-CORD-19 dataset and what (and how many) details are mentioned along in their article context. These extraction results are conditional on the extraction performance of our pipeline (Lopez et al., 2021b). About 50% of the extracted software mentions are solely names of the software without further details; 35% provide a version; 21% mention their publisher; and 9% have a URL given in the text.

### Stratified sampling

To get an overview of how software has been mentioned in recent publications, we took software mentions extracted from the 61,175 articles (87%) published since 2016 in the Softcite-CORD-19 dataset as our sample frame. This sample frame contains 250,163 extracted software mentions. Due to the time when COVID-19 emerged, the sample frame is skewed toward software mentions in publications after 2019; software mentions from articles published since 2020 totals 85% of the extracted mentions in our sample frame.

We systematically constructed a random sample of software mentions from our sample frame, stratified by the *impact factor* of the article’s publication venue as well as the article’s *mention density*.

#### Impact factor

Because a scientific reader’s attention is often concentrated on a few publication venues (Bradford, 1934; Brookes, 1985), these venues may have an outsized effect on their perception of the scientific reporting practices. The journal impact factor, developed by the Institute of Scientific Information (ISI) and annually calculated and published by the ISI Web of Science (WoS), is calculated based on the citations to one journal within a given period of time. Because citation to one journal is an outcome of one’s scientific attention, we used the journal impact factor as the proxy of collective scientific attention. We matched the 6,997 distinct journal titles in the Softcite-CORD-19 dataset (version 0.2.1) using CrossRef DOI metadata to the 12,312 indexed journals in the ISI WoS 2020 Journal Citation Report. 78% of the publications (N = 47,959) in the sample frame were identified as articles from a venue with an indexed journal impact factor.

We then divided the publications in the sample frame into different strata based on the range that their journal impact factor ranking falls in: 1–10, 11–100, 101–1,000, 1,001–12,982, and a “No impact factor” group for those articles from venues without an indexed impact factor.^2^ 2There are also numerous preprints in the CORD-19 corpus.This stratification balanced the coverage of articles from journals that receive different levels of attention in our sample. This choice also enabled us to examine software citation practices in comparison with the prior analysis of 90 biological publications from 2000 to 2010 in (Howison & Bullard, 2016). In that study, 90 articles from journals indexed in the 2010 ISI WoS biology-related subject headings were randomly sampled by a 3-tier journal impact stratification: journals with impact factor ranked 1 through 10, then those ranked 11–100, finally those ranked 111–1,455.

#### Mention density

**Figure 2:**
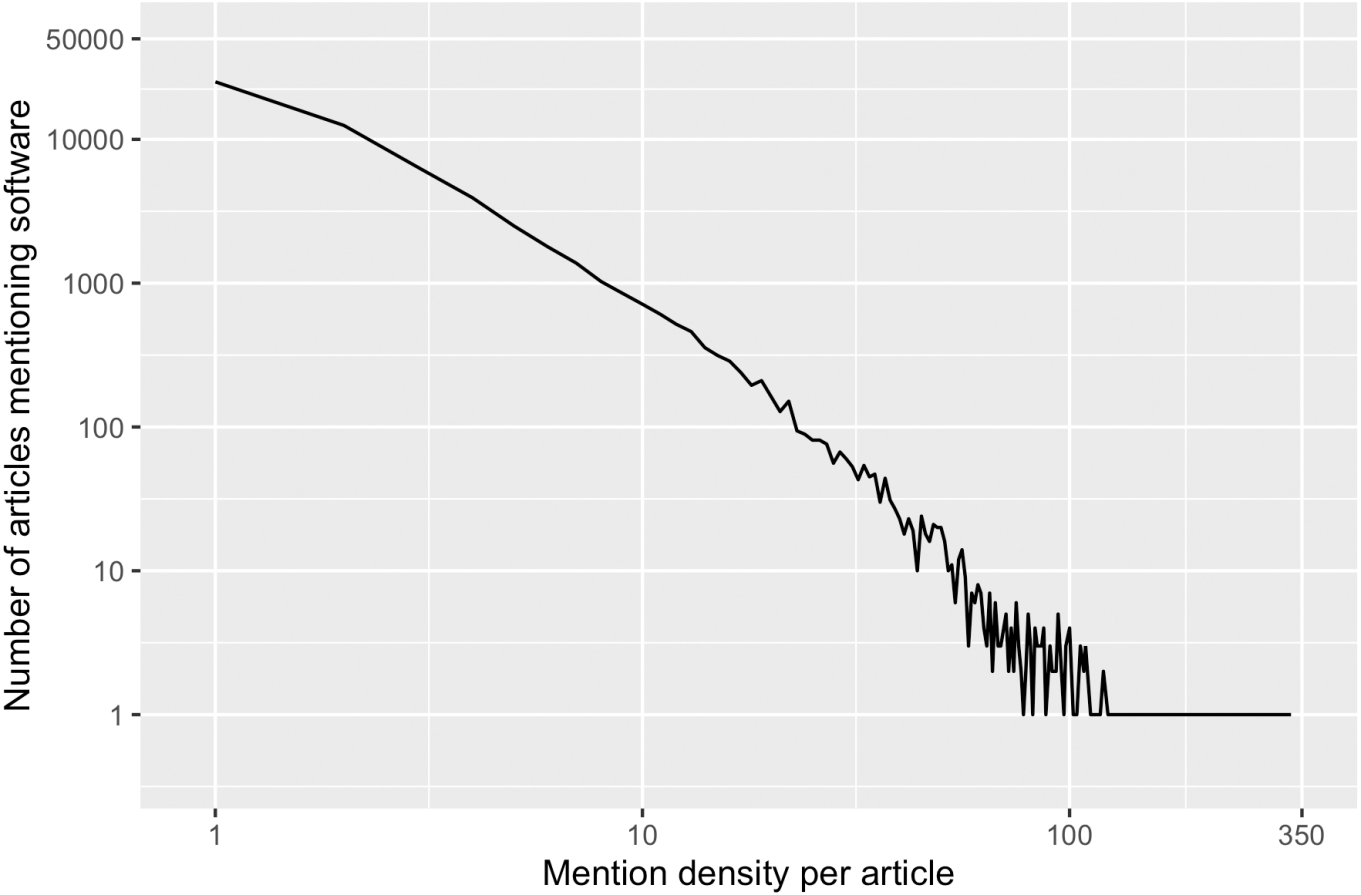
In our sample frame, number of articles mentioning software varies by mention density per article. 25,116 articles in the sample frame (41%) had only one software mention, while one particular article had the most software mention (*N* = 330). Only 11% of the articles in the sample frame had more than 9 software mentions.

The number of software mentions extracted per article, *i.e.,* the *mention density*, varied significantly in the sample frame. The average mention density is 4.1 with a standard deviation of 7.2, and ranges from one to 330 mentions per article. In Fig. 2, this variance in our sample frame is further illustrated. Because of this variation, we also stratified our sample by mention density.

**Figure 3:**
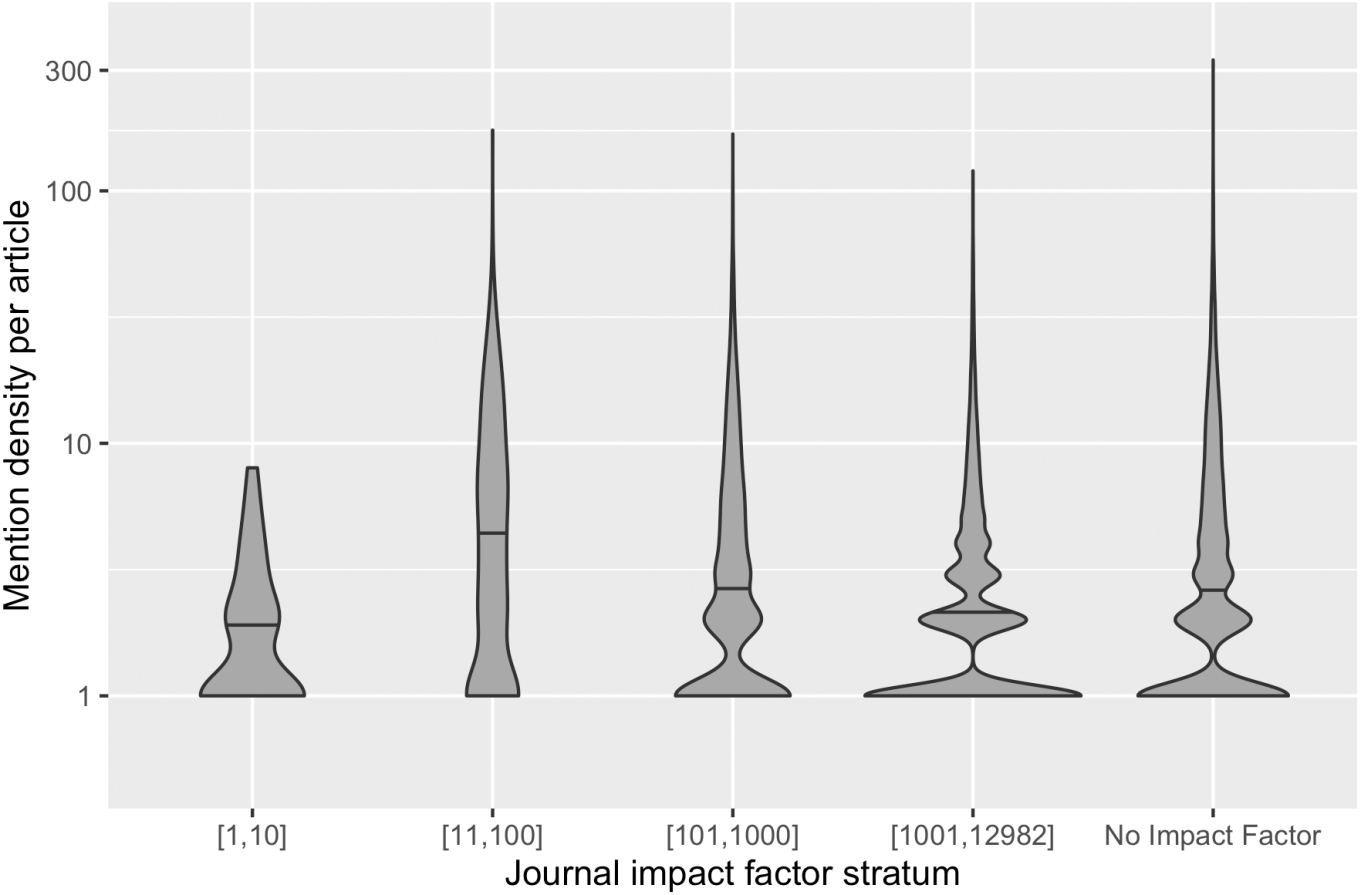
The distribution of mention density in each journal impact factor stratum. The horizontal line within each violin plot denotes the median mention density in the distribution.

The variation of mention density across articles could be the result of changing practices of mentioning software over time, different genres of publications across venues and domains, requirements of journals, and/or writing conventions of authors, and so forth. Figure 3 demonstrates the distribution of mention density in different impact strata: Articles in the top impact factor stratum have a narrower distribution of mention density. Articles in lower/no journal impact factor strata have more articles with more than eight software mentions; these strata also have outlier articles where more than 100 software mentions are recognized. To ensure representation of these different distributions, we grouped the articles in our sample frame into three subsets by their mention density. The resulting sample frame and the number of articles in each stratum are summarized in Table 1, which also shows the uneven distribution of articles across different strata. Our stratification therefore supports more balanced sampling.

**Table 1:** Number of articles and their percentage in the sample frame from each sample stratum.

**Mention density impact strata**	**[0, 1]** (41.06%)	**[2, 8]** (48.13%)	**[9, 350]** (10.82%)
[1, 10] (0.05%)	17	16	0
[11, 100] (0.95%)	179	259	142
[101, 1,000] (9.31%)	2,201	2,708	784
[1,001, 12,982] (51.14%)	13,606	15,291	2,385
No Impact Factor (38.56%)	9,113	11,168	3,306

We examined annotation results in each stratum after annotation. However, we did not find substantial differences across any strata, so we do not report results broken down by these strata in this article.

We randomly sampled 15 articles from each stratum. Because there is no article in the stratum with the highest mention density ([9,350]) and highest journal impact rank ([1,10]), we skipped this stratum and obtained a sample of 210 articles from the remaining 14 strata. Next, we randomly sampled one software mention from all the extracted mentions of each article. Given that the CORD-19 dataset is a somewhat noisy corpus, when we encountered an article that is not a research article (*e.g.*, scientific news postings), we moved to the next article in the same sample stratum and randomly sampled an extracted mention from this article to replace the original one. Accordingly, we gained a sample of 210 extracted software mentions. The scripts for implementing this stratified sampling procedure as well as all the analyses and figures discussed in the following sections can be found in https://github.com/caifand/cord19-sw-analysis.

### Annotation

We developed a coding scheme to manually validate and annotate the sampled extraction results. Based on empirical descriptions of software citation practices and recommendations from advocacy, this coding scheme allows for capture of current practices for software citation and comparison to advocated-for best practices (Smith, Katz & Niemeyer, 2016; Katz et al., 2019; Hong et al., 2021). The full coding scheme contains 57 codes. Some codes were annotated based only on the content of the mention and its original article, while others, such as those regarding the access and archiving status of software, required annotators to conduct web searches and locate online presences of the mentioned software.

The first codes in our scheme validate the extracted mentions and their details. These codes require examination of the extracted mention content (sentences from original full-texts that mentioned software) and their accompanying bibliographical items. The original PDF publications were also examined to confirm whether the extracted results were consistent with the source article. For any problematic software extraction results, we manually corrected the extraction and annotated them accordingly. If it was not found in the corresponding PDF, we randomly sampled another mention from the same article, or moved to the next article in the sample frame and randomly sampled one of its mentions if the original article had only a single extracted mention. Throughout the sample annotation process, we found 5% of the automatically extracted software mentions are false positives (95% CI [0.03, 0.09]).

**Figure 4:**
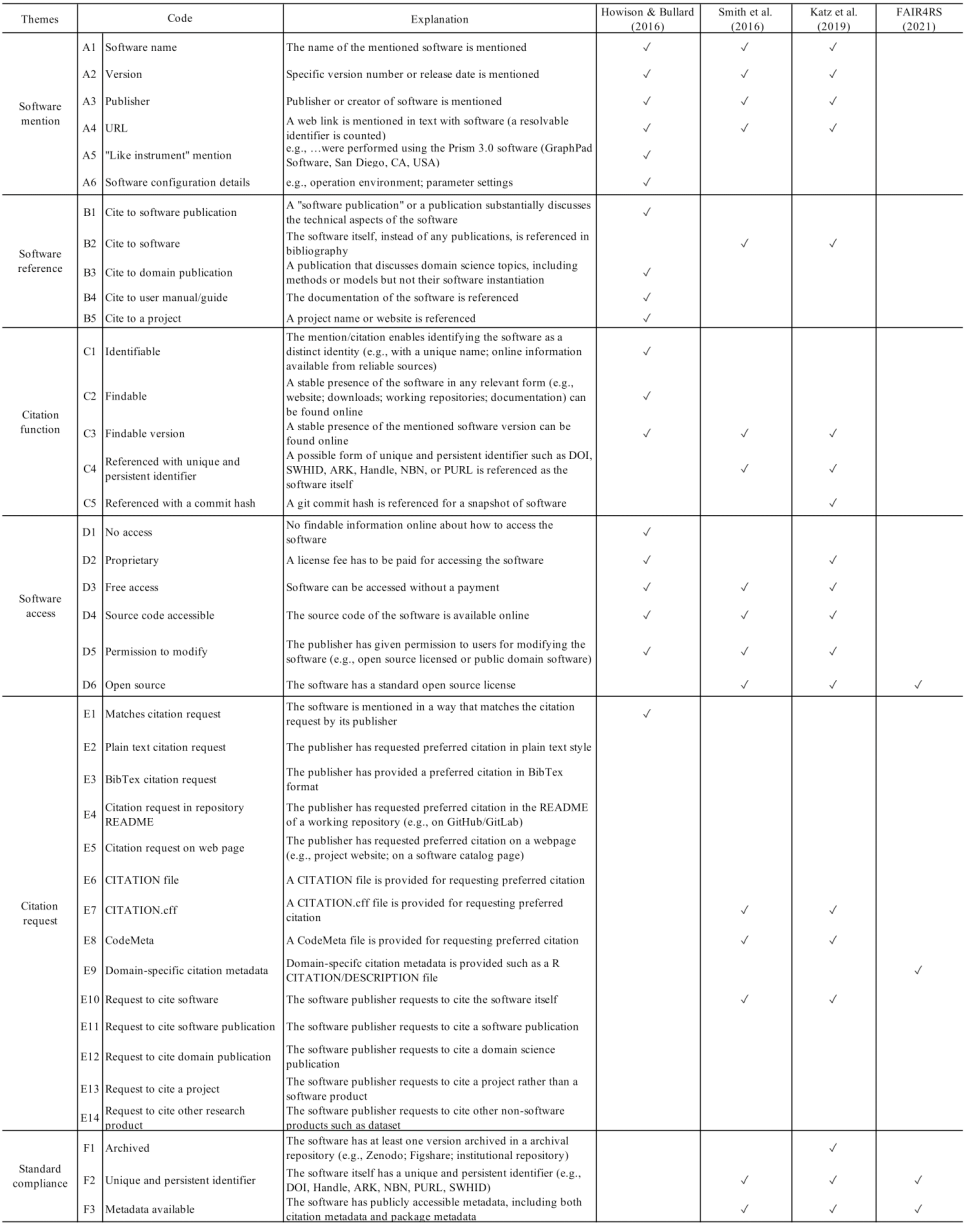
A crosswalk between the software extraction coding scheme and advocacy recommendations.

Figure 4 presents the remainder of our codes. We first replicated the coding scheme applied by Howison & Bullard (2016), including codes about the in-text software mentions and their bibliographical entries, the functions of these software mentions, the access to the mentioned software, and whether the software citation aligns with a discoverable citation request (codes A1–B1, B3–C3, D1–D5, & E1). We then added codes E2–E14 to specify the format, contents, and location of citation requests. Next, we identify whether these codes meet the advocacy recommendations for software citation, particularly those discussed in Smith, Katz & Niemeyer (2016) and Katz et al. (2019). This mapping from empirical descriptions to advocacy recommendations allows us to compare annotated practices with advocacy recommendations.

Here we explain how the codes are mapped from the scheme of empirical descriptions (Howison & Bullard, 2016) to specific advocacy recommendations, as the crosswalk in Fig. 4 shows. For example, if a software mention includes the name and version of the software, credits its creator(s), and provides a URL to facilitate the access (codes A1–A4), then the basic requirements of the Software Citation Principles (Smith, Katz & Niemeyer, 2016) are met. If the mention enables the access to the software, no matter it is open source or closed source, then it further conforms with later recommendations (codes D2–D5; particularly, Katz et al. (2019) have specified citation expectations for closed source software as the code D2 concerns). If a mentioned piece of software has an available citation request online for the mentioned software and it requests to cite the software artifact rather than any other complementary artifact (such as a publication or user manual) and if specific formats of citation metadata (*e.g.*, CITATION.cff, CodeMeta) are adopted, these practices also comply with the advocacy recommendations (codes E7, E8, E10). In cases where a citation request is declared by domain-specific metadata, such as by an R DESCRIPTION/CITATION file, it does not conform with those software citation advocacy recommendations, but meets one of the FAIR Principles for Research Software (FAIR4RS) that the software metadata should be *findable*. While the FAIR4RS Principles are not primarily advocated for software citation implementation, we included them for reference.

To better examine the extent to which existing citation practices align with advocacy recommendations, we added codes that are primarily derived from advocacy recommendations (codes B2, C4–C5, D6, F1–F3). These codes concern whether the software artifact itself is referenced in the bibliography with a unique and/or persistent identifier and whether the software artifact is archived, registered with an persistent identifier, or has available metadata online. These emphases from current advocacy recommendations ensure that the software artifact can be referenced like traditional research products such as scholarly publications. In particular, valid form of metadata available (codes E7–E9, F2 & F3) and/or a unique and persistent identifier make the software in accordance with the FAIR4RS “Findable” principle (codes F1, F2, and/or F3). If the software code follows standard open source (code D6), then it is “reusable” in the sense of the FAIR4RS definition (specifically, see FAIR4RS principle R1.1 in Hong et al., 2021). Given our emphasis on software citation, we considered only the aspects of FAIR4RS related to software citation.

To confirm the inter-annotator reliability of the coding scheme, two authors initially annotated a sub-sample (10% of the full sample). They achieved 93.3% percentage agreement across all the codes in the full coding scheme; discussions resolved the remaining disagreements. Later, a third author joined as an annotator using the validated coding scheme. Questions emerging from the annotation process were then discussed among annotators to reach consensus and refine the coding scheme accordingly. Finally, after the annotation was finished, one author wrote a script to validate the logical constraints and consistency between coding results, and re-annotated when violations of logical constraints were identified.

## Results

As our annotation scheme is extended from previous empirical descriptions of software citation practices, we are able to compare our results with earlier findings from Howison & Bullard (2016) to see if software citation practices have changed. The annotation scheme also allows us to compare existing practices with advocacy recommendations and understand whether they converge or deviate. In this section, we report the annotation results with these considerations. We first describe the forms that software mentions take in our sample and the functions of scholarly citation they are able to realize. Next, we discuss the extent to which the mentioned software is citable as the advocacy has recommended by examining their metadata availability, archiving status, and persistent identification. Finally, we present findings about how software citation has been requested by software creators and publishers. Our annotation was conducted in August 2021 and all the annotations about software online presences correspond to results of web searches conducted then. We report all the proportions of coded categories with 95% confidence interval. These results were calculated using the prop.test function in the base R package stats (R Core Team, 2019).

### How software is mentioned

#### Forms of software mention

**Figure 5:**
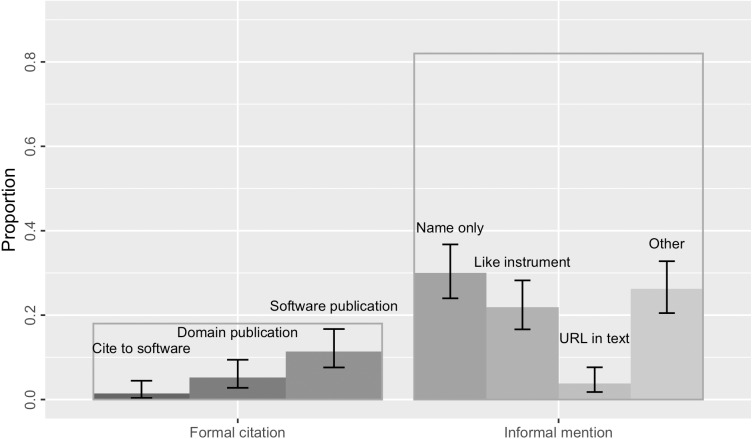
18% of the software mentions in the sample were formal citations (the transparent bar on the left) of either the software itself, a domain publication, or a software publication. The remaining 82% were informal mentions without bibliographical references, including those mentioning software name only, following a “like instrument” style, providing a URL in text, or other forms of casual mentions that might refer to a version and/or software creator. Error bars show the 95% confidence interval estimates.

Overall, 82% of the software mentions in our sample (*N* = 172; 95% [0.76, 0.87]) were informal without a bibliographical reference (Fig. 5). Most of these informal mentions only gave the name of the software; sometimes they additionally referred to a version, software creator or publisher, and/or a URL. 30% of the mentions (*N* = 63; [0.24, 0.37]) only referred to the software by its name (Fig. 5). Because our annotation procedures rely on the results of the software mention recognizer, it is possible that we have missed mentions that provided no name (*e.g.*, “using a program we wrote”). However, we do not expect these would have been substantial, given that Howison & Bullard (2016) only found 1% ([0, 0.04]) unnamed software mentions in their corpus.

Consistent with findings from Howison & Bullard (2016), we found software was sometimes mentioned “like instrument” (22% of the sample, *N* = 46, [0.17, 0.28]) in that it is similar to how researchers conventionally reference scientific instruments provided by vendors or manufacturers when authoring academic publications. This kind of mentions specify the software name, the name of its provider, and often the geographical location of the provider (*e.g.*, “STATA, StataCorp, College Station, TX”). 4% of the mentions (*N* = 9; [0.02, 0.08]) in the sample provided a URL in text for software access. These results are indistinguishable from the findings in Howison & Bullard (2016) that 19% ([0.14, 0.24]) of the software mentions identified in the biology literature sample were in the “like instrument” style and 5% ([0.03, 0.08]) gave a URL.

In contrast to findings from Howison & Bullard (2016), we did not find any software mentions citing a software manual or its project. The majority of formal citations still cited a publication—either a domain science publication (5%, *N* = 11, [0.02, 0.09]), or, more commonly, a software publication (11%, *N* = 24, [0.08, 0.17]). In Howison & Bullard’s sample, even more software mentions cited a publication (37%; [0.31, 0.43]).

Software Citation Principles (Smith, Katz & Niemeyer, 2016) suggest that the software artifact itself should be cited as a first-class research product; any relevant publications should be cited as companions. In this case, we found three formal citations of the software artifact in bibliography (1.4%; [0.004, 0.05]), which was not found in Howison & Bullard (2016), but none of them included an identifier such as a persistent DOI or a commit hash (Katz et al., 2019). Thus, none of these software mentions or citations met the Software Citation Principles’ goal of *unique identification* or *persistence*. The identification of the mentioned software, especially when a specific version is involved, mainly relies on whether there is a URL referenced in text, otherwise the academic readers would need to make use of any clue revealed by the software mention to look for the software.

#### Functions of software mentions

Traditional scholarly citation allows for the identification, access, and subsequently verifying and building upon the cited work (Howison & Bullard, 2016). Software citation advocacy seeks to enable these functions by recommending best practices for referencing software in scholarly work (Katz et al., 2021). Howison & Bullard (2016) found that informal software mentions could still function to some extent in scholarly communications. We assessed and annotated the enabled functions of mentions in our sample in accordance.

**Figure 6:**
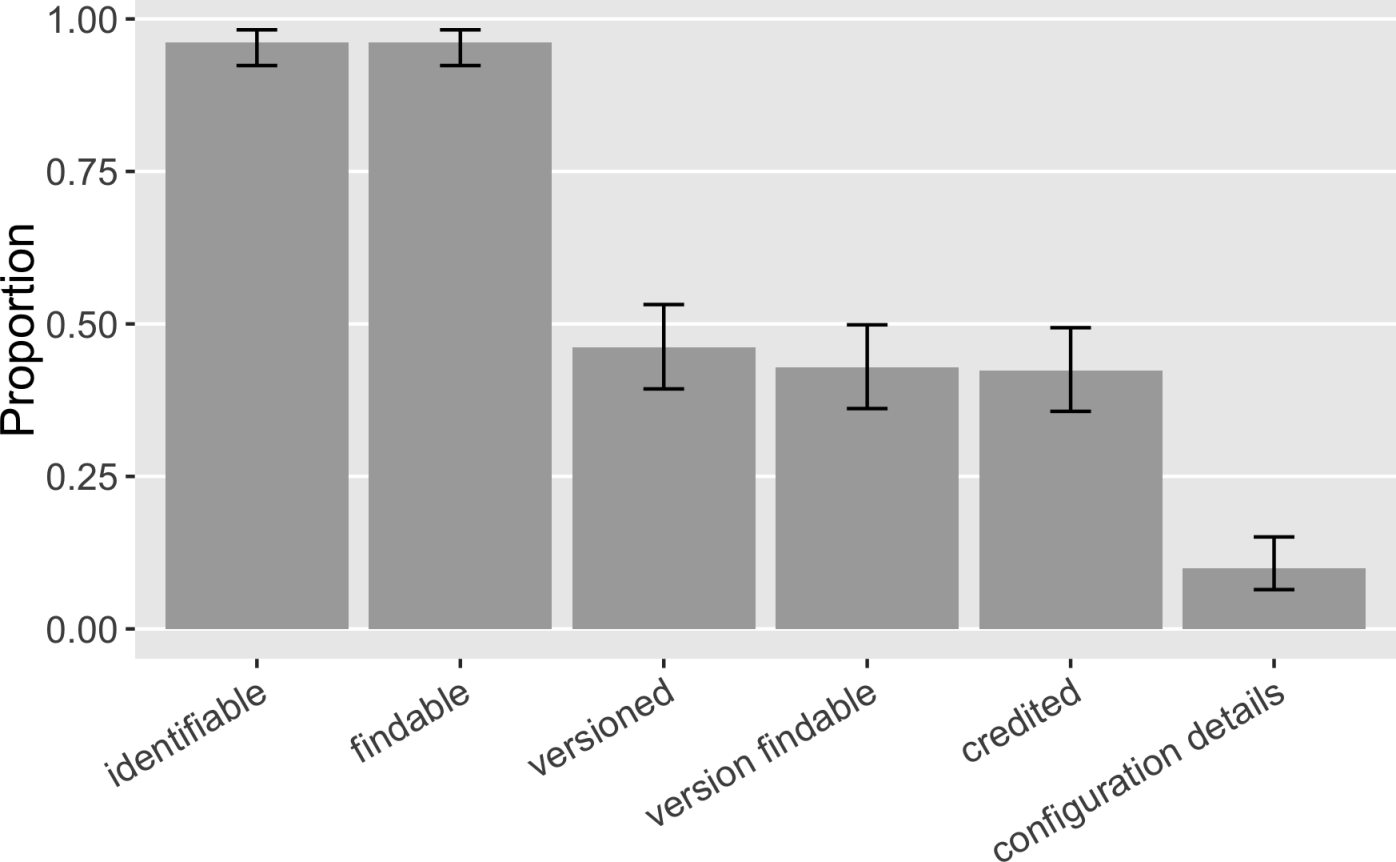
Functions of software mentions in our sample (error bars show 95% CI): Do software mentions enable the identification and discovery of software and its versions? Do software mentions credit its contributors? Do software mentions provide further configuration detail that facilitates reuse?

A software mention was annotated as “identifiable” if the information given distinguished the software as a distinct entity. It was then annotated as “findable” when the mention facilitated the online search and discovery of the software. 96% ([0.92, 0.98]) of the software mentions were both identifiable and findable, enabling the annotator to discover a distinct piece of software *via* web search (Fig. 6). These results suggest an improvement on findable software from Howison & Bullard’s results: they found 93% ([0.88, 0.96]) of software mentions supported the successful identification of software but fewer (86%; [0.80, 0.90]) enabled online discovery. Overall, it is positive that we were able to identify and find almost all of the software even though 82% of all the identified mentions were informal. This finding implies that most software has online presence(s).

**Figure 7:**
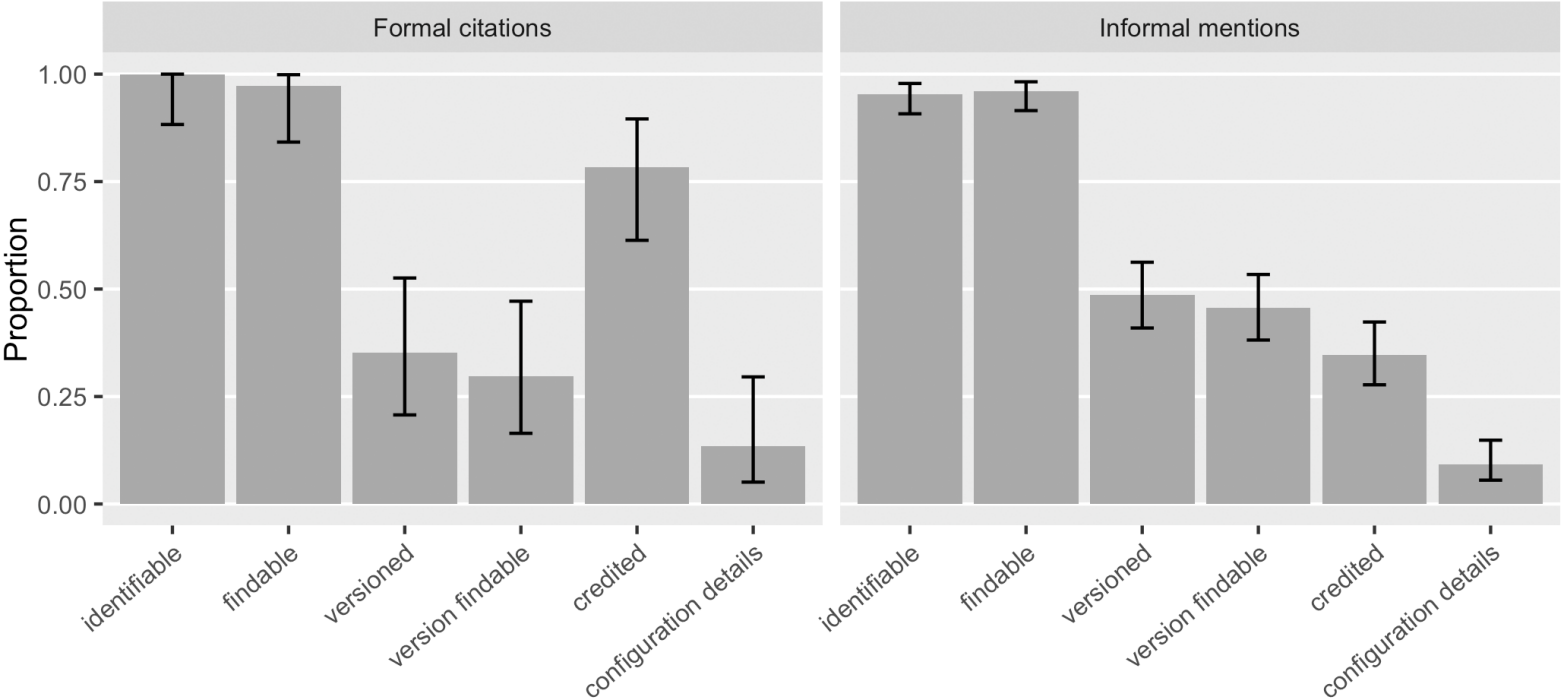
How do existing formal citations and informal mentions of software function? In our sample, informal mentions tended to provide more specific versions; and formal citations credited better. Most formal citations in our sample referenced a publication rather than the software artifact (Error bars show 95% CI).

We saw that 46% ([0.39, 0.53]) of the software mentions specified a version and 43% ([0.36, 0.50]) of the software mentions had a findable version online, an increase over Howison & Bullard’s findings (28%, [0.22, 0.35], and 5%, [0.03, 0.10], respectively). 48% ([0.41, 0.56]) of the informal software mentions and 35% ([0.21, 0.53]) of the formal citations identified specific versions (Fig. 7). 46% ([0.38, 0.53]) of the informal mentions led to findable versions online and so did the 30% ([0.16, 0.47]) of the formal citations. The annotation results also suggest that 78% ([0.61, 0.90]) of the formal citations and 35% ([0.28, 0.42]) of the informal mentions identified authorship and thus were able to give credit. We interpret a likely reason for the difference between existing formal citations and informal mentions in their strength to give credit and to identify versions: 92% ([0.77, 0.98]) of the formal software citations reference a publication, indicating clear authorship; but a publication, even a “software paper”, is not often version specific. Current software citation advocacy has also recognized this: the credit given by publication is often one-off and static, not sufficient to account for the dynamic authorship of the actual software work across versions (Katz et al., 2019; Katz & Smith, 2015).

**Figure 8:**
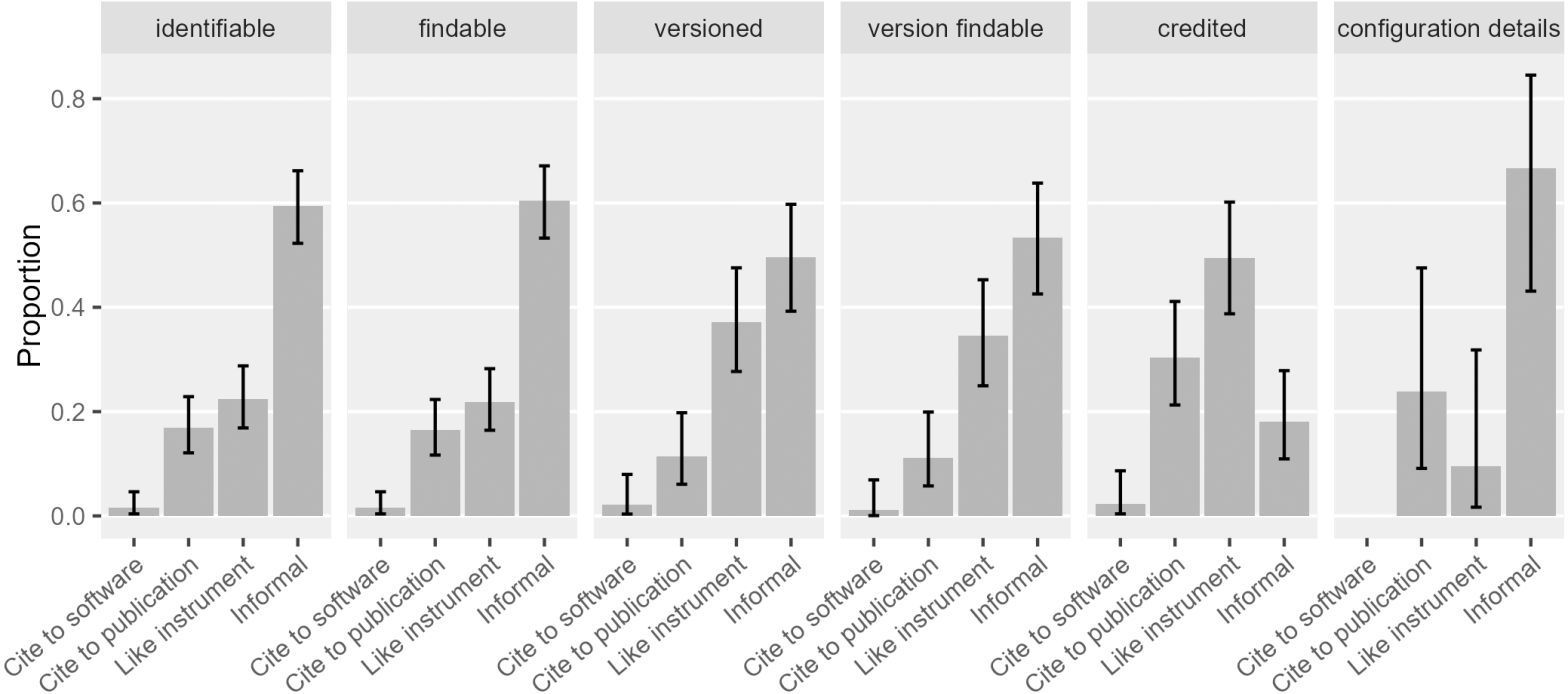
Types of software mentions that serve different functions. Software mentions that give credit were mostly driven by “like instrument” mentions and formal citations to a publication (Error bars show 95% CI).

Proper crediting has been a strong motivation for software citation advocacy. In our sample, 42% ([0.36, 0.49]) of the software mentions recognized the creators or publishers of the software. In contrast, Howison & Bullard (2016) found around 77% ([0.70, 0.83]) of the mentions recognized creators or publishers. This is possibly driven by the 37% of the software mentions in their sample that cited a publication while only 17% in ours did. When looking more closely (Fig. 8), crediting mentions in our sample were mostly “like instrument” mentions (49% of all the crediting mentions; [0.39, 0.60]), and secondarily, formal citation of articles (30% of all the crediting mentions; [0.21, 0.41]). As we will discuss in the later section, “like instrument” mentions mostly credited proprietary software publishers, who are in less need of scholarly credit. In cases of software citation, information about the proprietary software publisher probably provides more accountability for the software involved and thus contributes to the integrity of scientific communication.

Finally, 10% ([0.06, 0.15]) of the mentions gave some additional detail about the actual configuration of mentioned software, usually specifying an operation environment or parameter settings. Such detail could facilitate the verification of the scientific method employed and the reuse of mentioned software.

**Figure 9:**
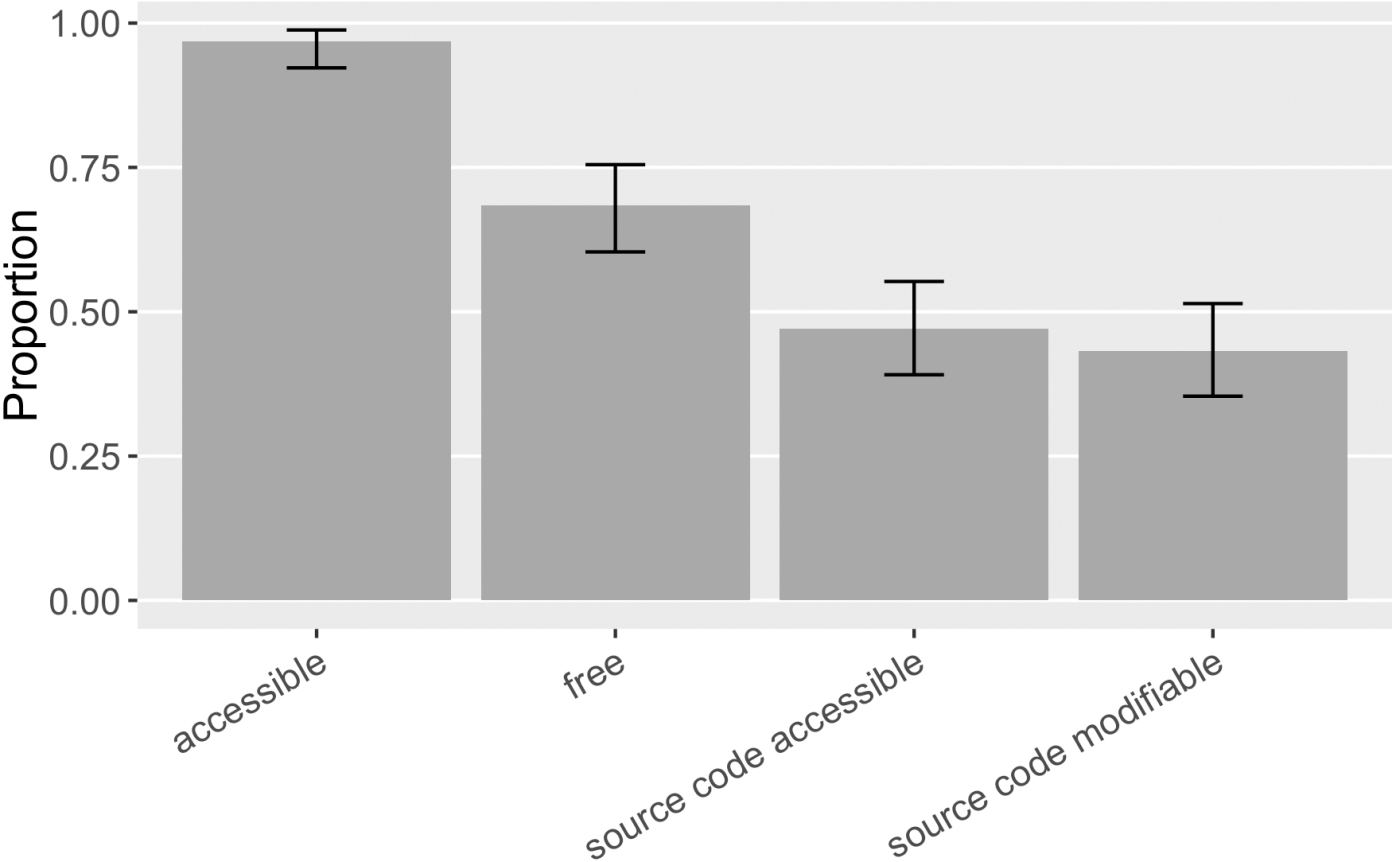
Accessibility of the software mentioned (error bars show 95% CI). Accessible software includes software with restricted or paid access. Free access software includes those with open source code available, or those in the form of executable or web services. Modifiable source code means the permission to modify the source code is formally granted.

Another key condition of reuse is whether the mentioned software is accessible and retrievable. Facilitating access is one advocated function of software citation (Smith, Katz & Niemeyer, 2016) as well as the conventional concern of scholarly citation (Howison & Bullard, 2016). For the 155 distinct pieces of software mentioned in our sample, 97% ([0.92, 0.99]) was accessible online, 68% ([0.60, 0.75]) of the mentioned software had free access, 47% ([0.39, 0.55]) had source code available, and 43% ([0.35, 0.51]) had both source code available and permission to modify it, such as an open source license, or a statement of waived copyrights (as the “source code modifiable” category in Fig. 9). In general, mentioned software in this sample was more accessible and actionable than that in Howison & Bullard (2016), where 79% ([0.71, 0.85]) of the mentioned software was accessible, 47% ([0.38, 0.56]) had free access, 32% ([0.24, 0.40]) had source code available, and 20% ([0.14, 0.27]) had modifiable source code.

### Software metadata, archiving, and identification

Advocates’ recommendations, such as Katz et al. (2019), recognize that sufficient citation metadata, software archiving, and unique and persistent identifiers are needed to cite the software artifact in a human- and machine-actionable manner (Smith, Katz & Niemeyer, 2016; Katz et al., 2019). These practices are essential for software and its citation in scholarly literature to become as visible as traditional academic publications, such as being identifiable and indexable by discovery tools. We therefore examined whether the 155 distinct pieces of software mentioned in our sample meet these advocacy recommendations.

Metadata availability was assessed by looking for publicly accessible metadata, including those primarily focused on software citation (*i.e.,* CITATION.cff (Druskat et al., 2021) and CodeMeta (Jones et al., 2017)) and metadata stored in public information registries^3^ 3We did not annotate metadata only associated with a minted DOI as the unique and persistent identifier category covers this possibility. Hence, we focus on other kinds of interoperable metadata.such as bio.tools (Ison et al., 2016). We also annotated the availability of language-specific software metadata such as R DESCRIPTION/CITATION files, Python setup.py files, and Java Maven pom.xml files, *etc.*, considering that these are interoperable with CodeMeta through crosswalks.

We searched for archival copies of mentioned software within Zenodo (European Organization For Nuclear Research and OpenAIRE, 2013), Figshare (https://figshare.com), and Software Heritage (Cosmo & Zacchiroli, 2017) with their within-site search feature. We considered searching institutional or domain-specific repositories but it is infeasible to create an exhausted list of repositories for manual search. We also experimented with using search engines like Google; it was neither effective to identify software archived in specific archival repositories.

We assessed the use of unique and persistent identifiers by searching for DOIs, ARKs (Archival Resource Keys), PURLs (Persistent URLs), and NBNs (National Bibliography Numbers). We chose this list to focus on globally used and interoperable identifiers. We considered other identifiers, including RRID, ASCL ID, and swMATH ID, some of which are very useful in specific domains; but limited annotation labor turned our focus on those globally used and indexed across systems of digital objects. Additionally, we did not include SWHID (Software Heritage ID) (Software Heritage Development Documentation, 2021) because those are automatically generated once it is archived in Software Heritage, already accounted for in our annotation about archiving.

**Figure 10:**
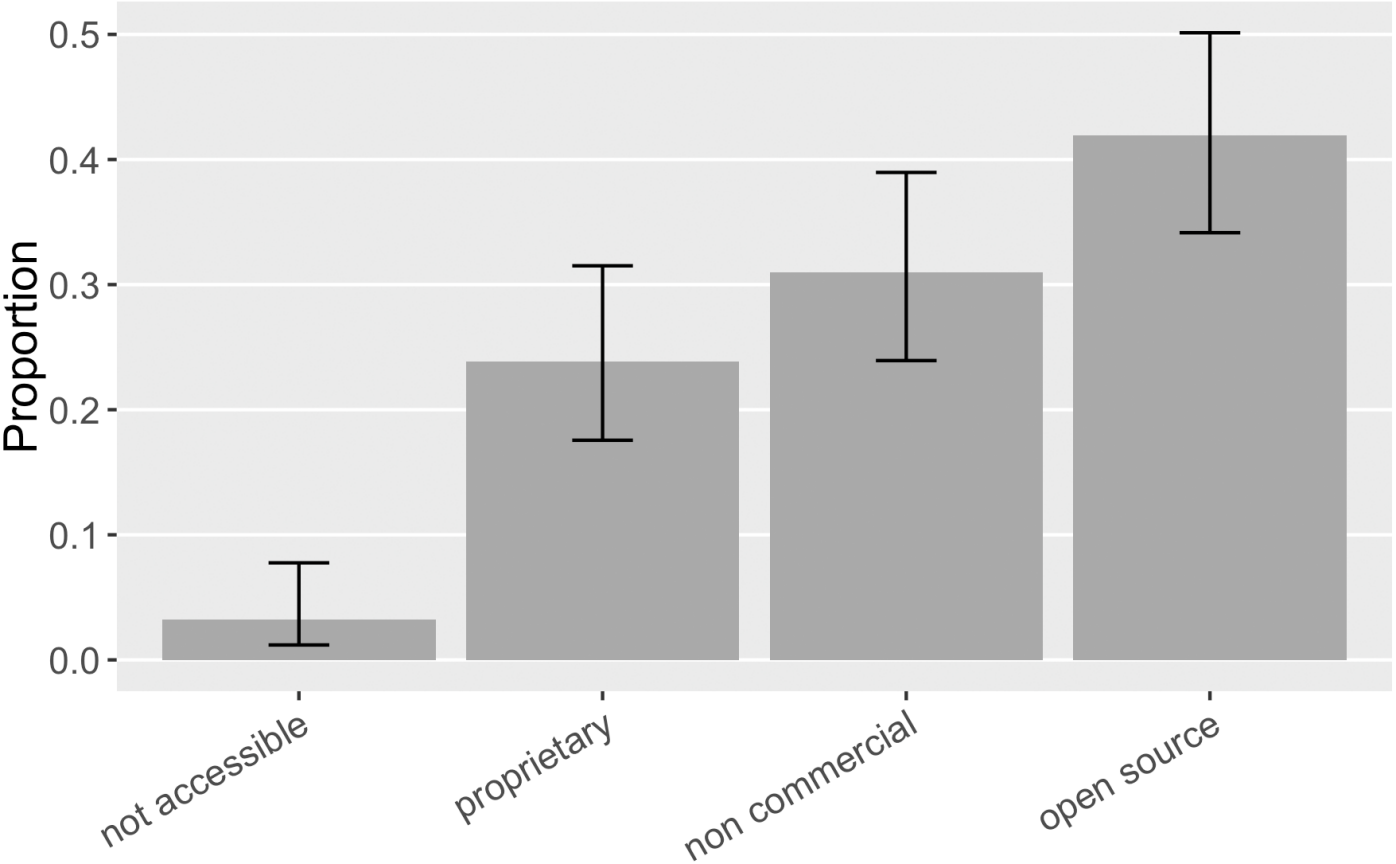
The property rights of the software mentioned (error bars show 95% CI). Five software mentions did not have an accessible record online, and their licensing status consequently could not be identified.

As the advocacy recommendation efforts recognize, unique and persistent identification, metadata accessibility, and archiving mechanisms can vary a lot between open source and closed source software (Katz et al., 2019). We thus examine the software in our sample with different property rights respectively. Particularly, we annotated closed source software with some kind of paywall as “proprietary”, software with a standard open source license as “open source”, and free access software without standard open source license as “non-commercial” (*e.g.*, public domain software or software with source code available but not following standard open source practices). As Fig. 10 shows, in our sample, five cases were not accessible and thus we cannot identify their licensing status; 24% ([0.18, 0.32]) of the mentioned software was proprietary while more was open source (42% of the mentioned software; [0.34, 0.50]); in-between is non-commercial software (31% of the mentioned software; [0.24, 0.39]). In Howison & Bullard’s sample, the corresponding proportion of proprietary, non-commercial, and open source software were 32% ([0.24, 0.40]), 27% ([0.21, 0.36]), and 20% ([0.14, 0.27]). Therefore, we observe a larger portion of open source software mentioned in this sample.

**Figure 11:**
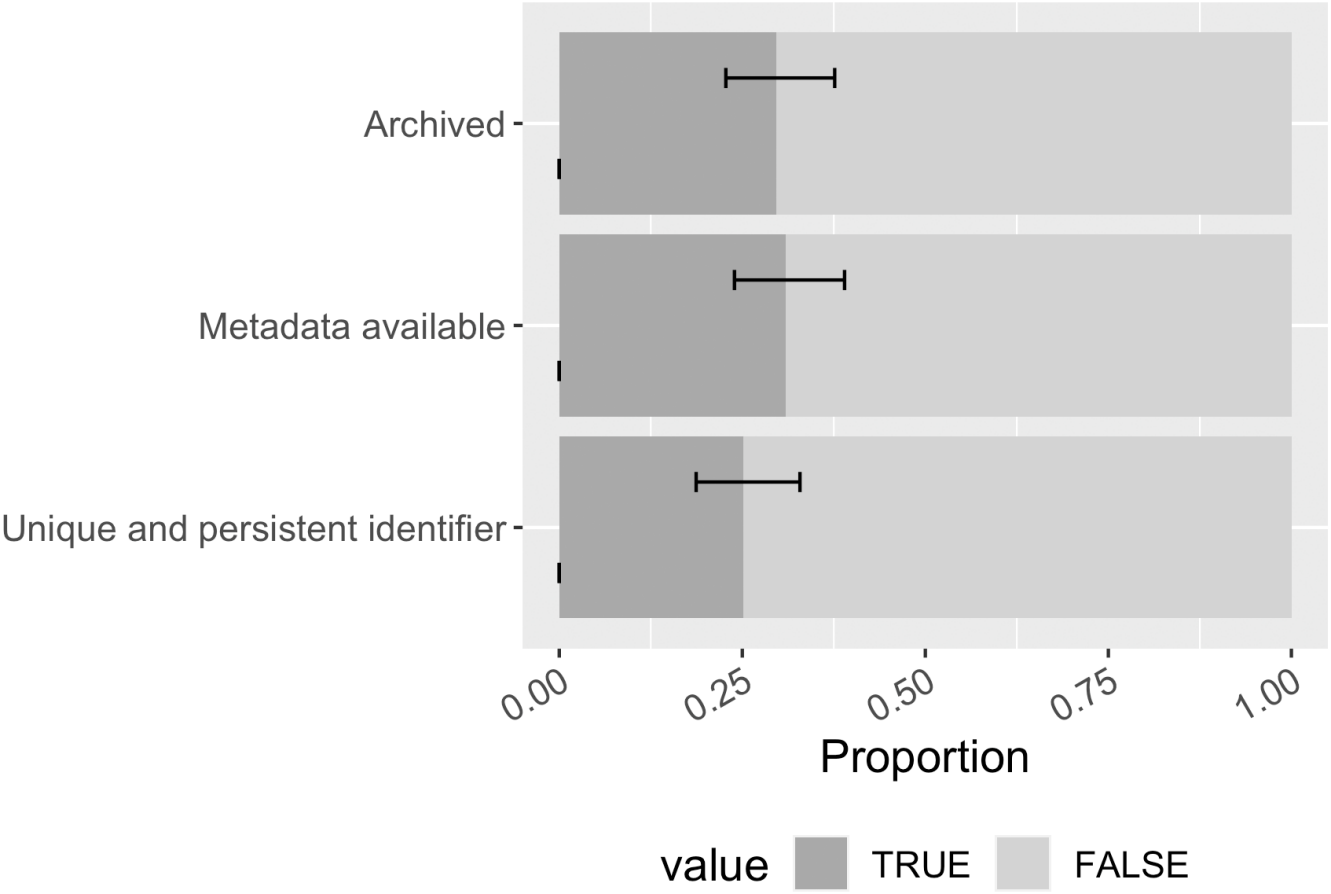
Software archiving status, metadata availability, and persistent identification status in the sample (error bars show 95% CI).

Overall, 30% ([0.23, 0.38]) of the mentioned software had at least one archived copy within Zenodo, Figshare, or Software Heritage (Fig. 11). While archiving in these repositories generates interoperable metadata for the archived item, slightly more software in the sample had publicly available metadata (31% of the mentioned software; [0.24, 0.39]), implying some software had metadata created elsewhere. 25% ([0.19, 0.33]) of the mentioned software had at least one unique and persistent identifier found among possibilities of DOI, ARK, PURL, and NBN; the most found identifier is DOI.

**Figure 12:**
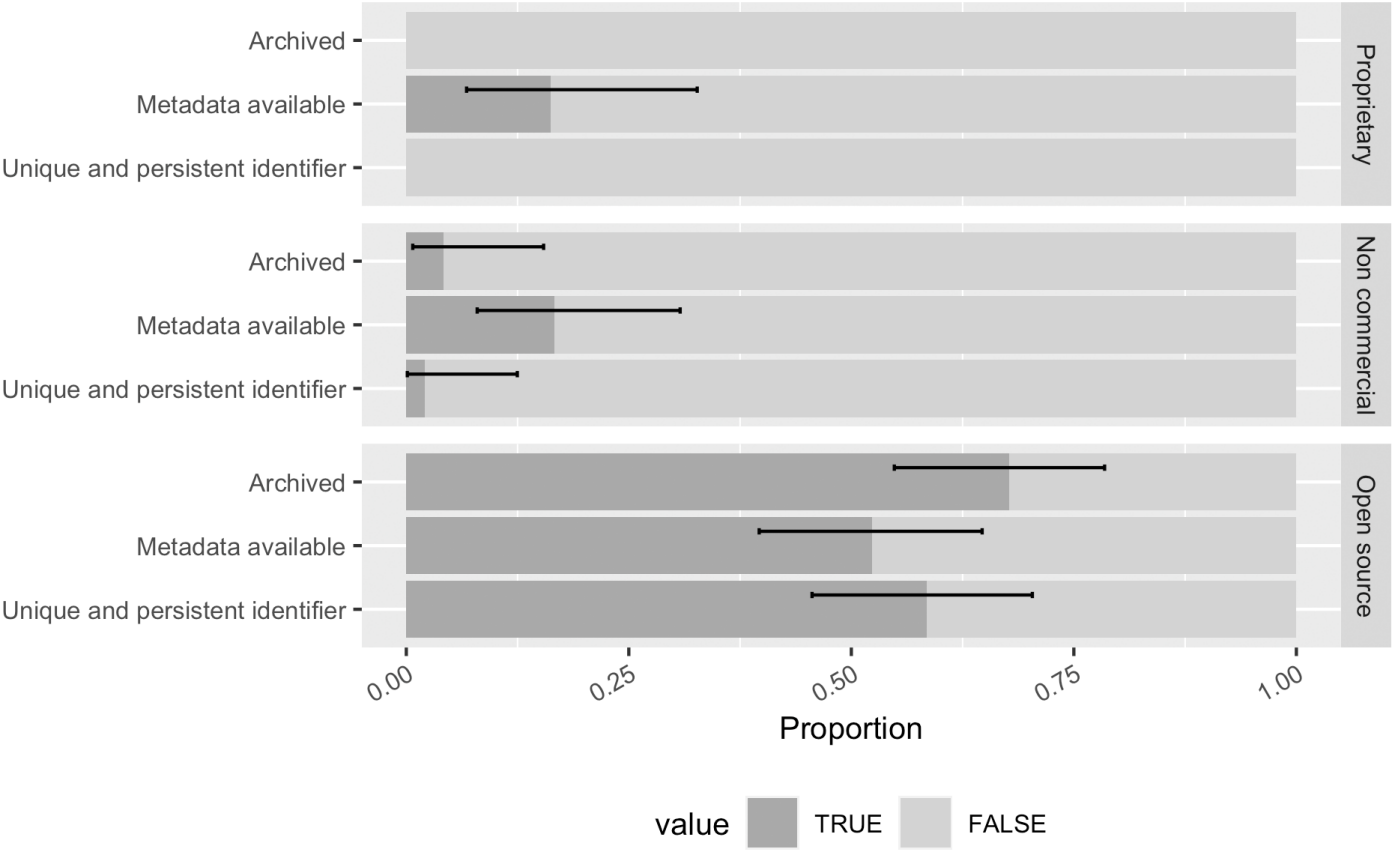
The archiving, metadata, and persistent identification status of software with different property rights in the sample (error bars show 95% CI). More than half of the open source software was archived and had available metadata and a persistent identifier. Proprietary software in the sample did not have any identified archival copies or persistent identifiers.

When examining across software with different kinds of property rights, we found that 68% ([0.55, 0.78]) of the open source software in the sample was archived in one of those aforementioned repositories (Fig. 12). During annotation, we noticed that this is largely driven by those software projects with a GitHub repository archived in the Software Heritage. Some non-commercial software in our sample with a source code repository on GitHub was also found archived by the Software Heritage. Only 4% of the non-commercial software was archived while no proprietary software in the sample was found archived. Although it is in theory possible to archive proprietary software in a closed source archive and only expose the unique identifier and metadata for discoverable records.

We have found publicly available metadata for 16% ([0.07, 0.33]) of the proprietary software in our sample, mostly stored in a public registry (*e.g.*, bio.tools). 17% ([0.08, 0.31]) of the non-commercial software had publicly accessible metadata, so as 52% ([0.40, 0.65]) of the open source software. But these available metadata are not oriented to citation. We found no CITATION.cff or CodeMeta in our sample.

Open source software was more likely than non-commercial software to have a unique and persistent identifier (58%, [0.46, 0.70] *vs.* 2%, [0.001, 0.12]); proprietary software had none at all.

Taken together, slightly more than half of the open source software in our sample met advocacy recommendations for citation. Closed source software, including non-commercial software without open source code available and proprietary software, indeed rarely have a unique and persistent identifier neither being frequently archived. It is not surprising given that archiving a piece of software is the primary way to obtain a unique and persistent identifier. The lack of the persistent identifier perhaps chiefly reduces the indexing potential of the closed source software. It is still valuable to reference proprietary software used for research in manuscripts especially when its routines constitute part of the research procedures.

### How citation is requested

Past research has shown that software creators and publishers seek scholarly credit by having their software cited (Howison & Herbsleb, 2011). Howison & Bullard (2016) found 18% ([0.13, 0.30]) of the mentioned software in their sample made a specific request for their software work to be cited, usually in the online presences of the software such as on a project web page. Bouquin et al. (2020) also looked for the preferred citations requested by software projects in their sample, motivated by examining the quality of these sources of citation information. Findings by these studies suggest that public citation requests do not necessarily prioritize the software artifact itself. Instead, software authors may request citations reference a software publication because those publications are more immediately compatible with the current system of scholarly citation, reputation, and impact.

**Figure 13:**
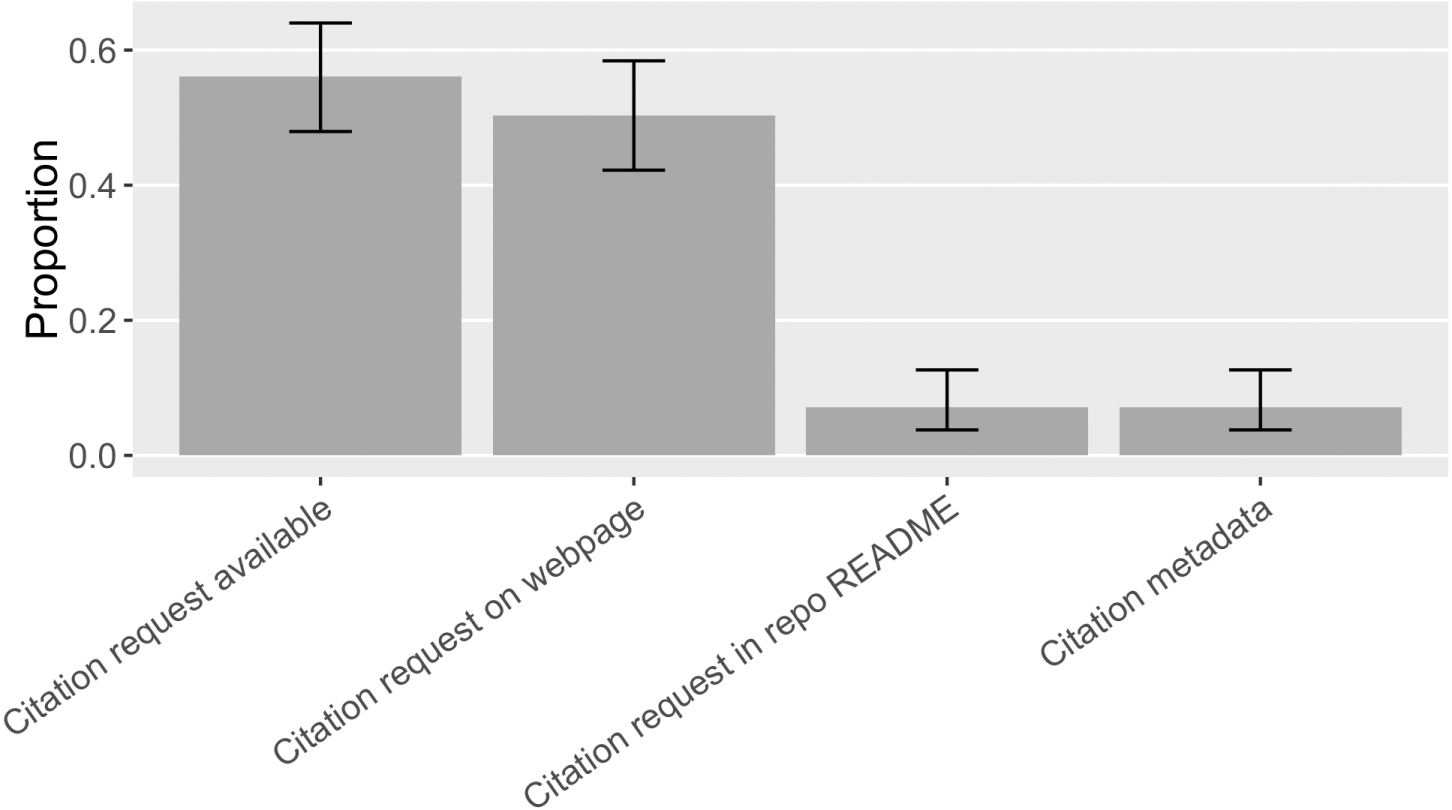
Locations of software citation request in the sample (error bars show 95% CI). Citation metadata includes formats like CITATION file and domain-specific citation metadata such as R CITATION/DESCRIPTION. No CITATION.cff or CodeMeta was found.

We are interested in understanding how common citation requests are, how software projects make specific citation requests, to what extent they orient researchers’ citation behavior, and whether they conform to the best practices recommended by the software citation advocacy. We therefore annotated the format and location of citation requests and what citation target was requested by searching for specific software’s citation request and looking through the online presence(s) of the software in our sample. Overall, we found 87 out of the 155 pieces of software (56%, [0.48, 0.64]) had at least one citation request findable online (Fig. 13); but only 13% ([0.09, 0.19]) of the sampled mentions followed these citation requests. Howison & Bullard (2016), in comparison, found 18% ([0.13, 0.30]) of the software in their sample made citation requests and 7%([0.04, 0.11]) of the software mentions followed these citation requests.

**Figure 14:**
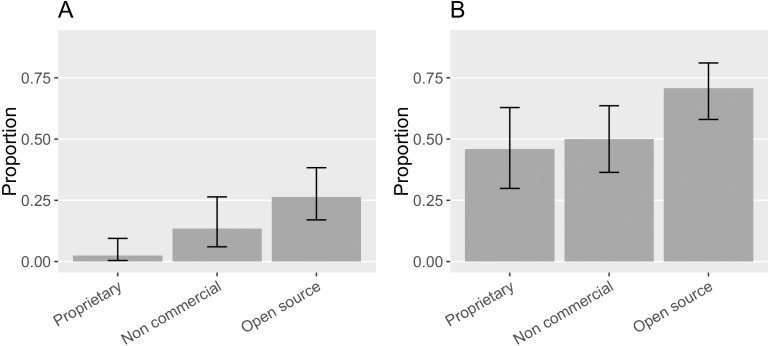
(A) Proportion of the mentioned software with different property rights in the sample that had citation request(s). (B) Proportion of the mentions of software with different property rights that matched the citation request (Error bars show 95% CI).

While we found an increase in citation requests and citations that follow them over Howison and Bullard’s findings, citations that follow recommendations were still fairly limited, varying by the type of software in question. 46% ([0.30, 0.63]) of the proprietary software in our sample had at least one citation request while 2% ([0.004, 0.09]) of the proprietary software mentions matched the requested citation. Half of the non-commercial software (50%, [0.36, 0.64]) in the sample requested software citation; 13% ([0.06, 0,.26]) of the non-commercial software mentions matched the actual citation request. 70% ([0.58, 0.81]) of the open source software made citation request and 26% ([0.17, 0.38]) of their mentions matched the request (see Fig. 14). In general, open source software projects made more citation requests, and their requests were more followed.

#### Locations of citation requests

Half of the software in the sample (50%, [0.42, 0.58]) had a preferred citation specified on a web page, mostly located on a software project website. Occasionally, we found a software catalog online suggesting a citation for its software entries (*e.g.*, Bioconductor (https://www.bioconductor.org/) and ASCL (Nemiroff & Wallin, 1999) do so). 7% ([0.04, 0.13]) of the software requested software citation in the README file of a source code repository. Another 7% ([0.04, 0.13]) had a metadata file available providing needed information for citation, especially the domain-specific format R CITATION/DESCRIPTION was found to specify a citation request (Fig. 13).

#### Formats of citation requests

**Figure 15:**
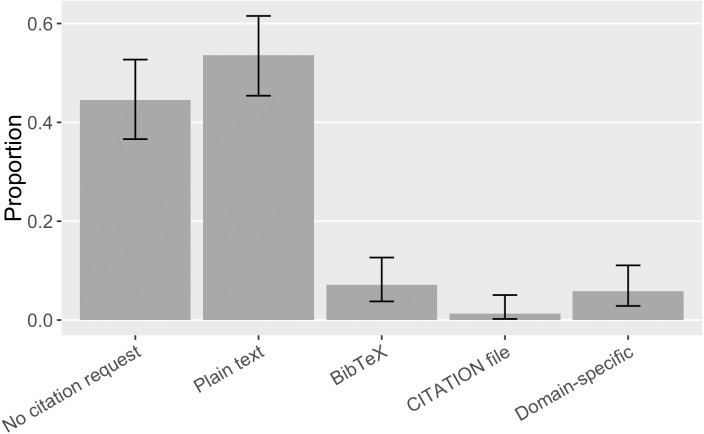
Formats of software citation requests in the sample (error bars show 95% CI).

Software projects in our sample use different formats to make citation requests. It was most common (54%, [0.45, 0.62]) to give a suggested citation in plain text format so that users can copy and paste it into their manuscript (Fig. 15). The next most popular format was a BibTeX formatted citation entry that can be used in the LaTeX document preparation system; but, at 7% ([0.04, 0.13]) of our sample, even this second-most-poplar citation request format was rare. Domain-specific citation metadata format, exclusively the R CITATION/DESCRIPTION file, was used by 6% ([0.03, 0.11]) of the cases in our sample; considering that it is a common expectation of the R language community, it may be a convenient choice for software developers to make a citation request. Finally, two pieces of software in our sample (1%, [0.002, 0.05]) used a CITATION file. These were initially embraced by advocates for crediting research software work (Wilson, 2013); because CITATION files are not machine-readable, advocates now prefer structured citation metadata like Citation File Format (CFF) and CodeMeta (Katz et al., 2019). We did not find any CITATION.cff or CodeMeta.json files, indicating these newer approaches have yet to replace older ones.

#### Objects of citation requests

If a request was made, it was the most common to ask that users cite a software publication (32%, [0.25, 0.4]). 20% ([0.14, 0.27]) of the software in our sample requested to cite the software artifact itself, in accordance with advocates’ recommendations (Smith, Katz & Niemeyer, 2016). 12% ([0.07, 0.18]) of the software in the sample requested that a domain science publication be cited. Two software projects requested the project itself be cited. One project requested that a dataset be cited; in this specific case, the software is the byproduct of the dataset.

**Figure 16:**
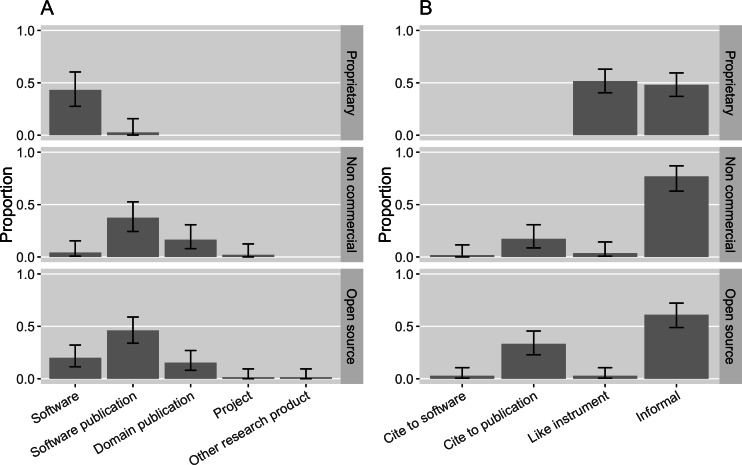
(A) What software projects with different property rights request to cite in the sample. (B) How software with different property rights is mentioned in the sample (Error bars show 95% CI).

It is worthwhile to look closely at these citation requests with respect to different software property rights because citation preferences vary (Fig. 16A). Non-commercial and open source software seemed to prefer a publication citation the most: 38% ([0.24, 0.53]) of the non-commercial software in the sample requested to cite a software publication while 46% ([0.34, 0.59]) of the open source software requested so; 17% ([0.08, 0.30]) of the non-commercial software and 15% ([0.08, 0.27]) of the open source software requested that users cite a domain science publication. 4% ([0.007, 0.15]) of the non-commercial software and 20% ([0.11, 0.32]) of the open source software requested that the software itself be the target of the citation.

In contrast, only one proprietary software project in our sample requested that users cite a software publication (2%, [0.001, 0.16]); 43% ([0.28, 0.60]) of the proprietary software requested that authors cite the software itself; and the remaining 46% ([0.30, 0.63]) of the proprietary software in the sample did not have a citation request available. This is likely because proprietary software publishers have more incentives to promote their software product but have less motive to engage in publishing about the science relevant to their software. Meanwhile, for open source and non-commercial software projects, citing their publication can more effectively demonstrate their scientific impact in the way most relevant to those evaluating their careers.

We accordingly examine how the software with different kinds of property rights in our sample is actually mentioned in publications (Fig. 16B). None of the mentions of proprietary software in our sample came with any bibliographic references. Most commonly they were referenced in the “like instrument” style (52%, [0.41, 0.63]), following the practices of referring to scientific instruments and their vendors. Yet, both informal and “like instrument” mentions ensure that the actual software artifact is referenced in publications. 17% ([0.09, 0.30]) of the non-commercial software mentions and 33% ([0.23, 0.46]) of the open source software mentions cited a publication; both were rarely mentioned like instruments in our sample: Only 4% ([0.006, 0.14]) of non-commercial software mentions and 3% ([0.004, 0.10]) of open source software mentions were instrument-like. Despite frequent requests to cite publications, 78% ([0.63, 0.87]) of the non-commercial software mentions and 61% ([0.49, 0.72]) of the open source software mentions were informal. Although 20% ([0.11, 0.32]) of the open source software in our sample requested that users cite the software artifact, only 3% of the open source mentions did so.

### Comparison with prior findings

**Figure 17:**
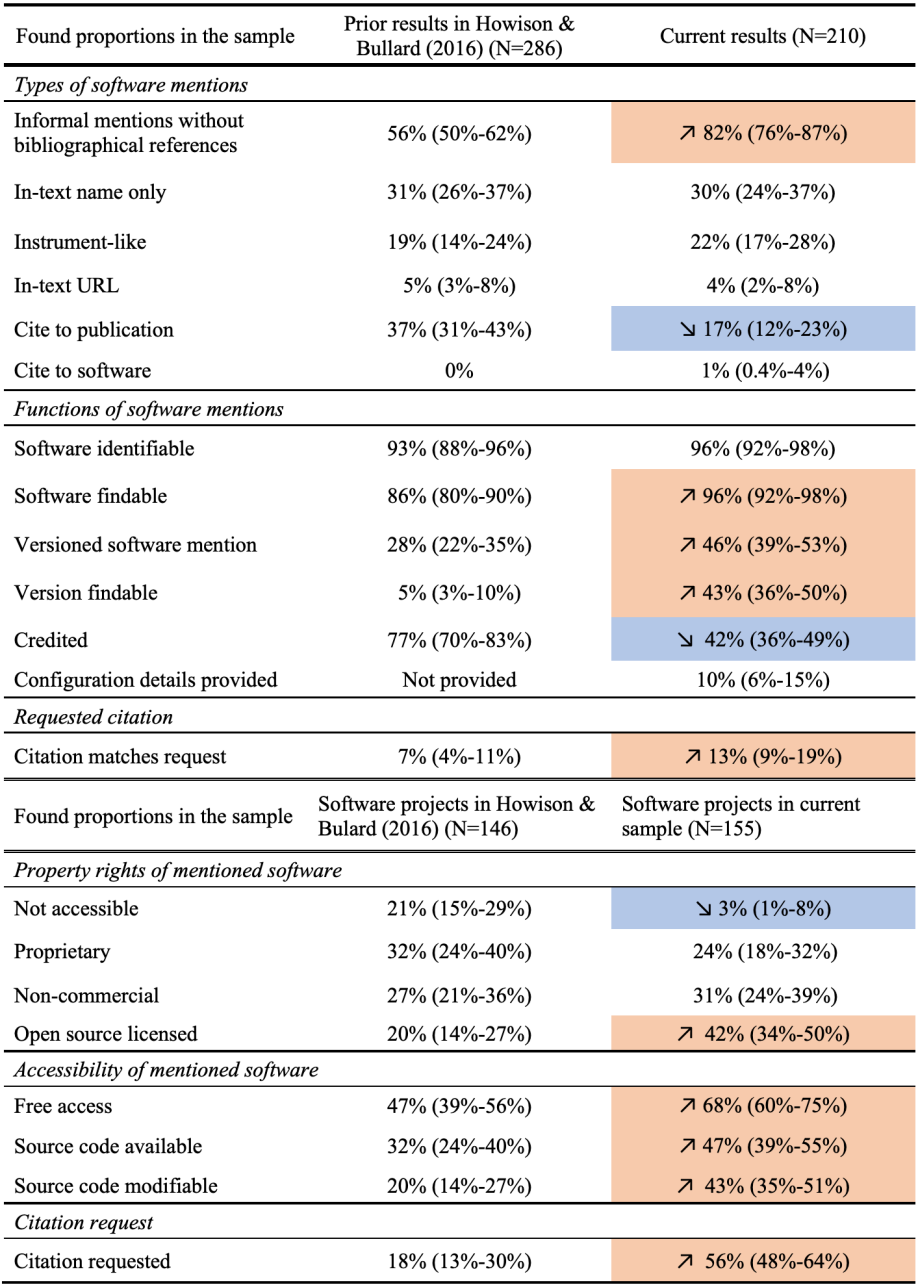
Comparing current results with previous findings by Howison & Bullard (2016). Statistically significant differences are highlighted in colors, with orange denoting an increase and blue denoting a decrease, correspondingly.

Finally, in Fig. 17 (on page 24), our annotation results are summarized in comparison with results from Howison & Bullard (2016). Given that the two sets of findings come from two independent samples, we conducted a two-sample significance test to compare the proportions calculated from annotations, using the prop.test function (Newcombe, 1998) in base R. When the difference between the proportion values across the two samples is tested as significant with *p* < .05, we have more statistical support to conclude a notable change. These statistically significant changes are highlighted in Fig. 17. The *In-text URL* and *Cite to software* categories have too few positive results (both under ten) to be an adequate sample for such significance testing.

Overall, we found more informal mentions and less citations to publications for referencing software in our sample. The mentioned software were more findable and more frequently referenced with a specific version, and these versions were also more accessible. However, proper crediting of software contributors did not seem to have improved. We see that mentioned software were more accessible; more mentioned software followed standard open source practices and provided modifiable source code. An increasing amount of software requested a preferred citation, but the actual mentions found did not always follow these requests.

## Discussion

In this section, we assess the limitations of our findings, discuss explanations for why we found the changes that we found, and discuss challenges for advocacy we perceived through our study.

### Limitations

In this paper, we compare our findings with those from previous publications, but our ability to compare and ascribe differences to changes in practices over time is subject to limitations. The sample in Howison & Bullard (2016) was taken from articles published between 2001 and 2010 in journals indexed in biology-related subject headings by the Web of Science; the sample in this study was constructed from papers published since 2016 from the CORD-19 corpus, covering perhaps more diverse venues that include many biology journals but only including content topically relevant to coronaviruses. Further, Howison and Bullard randomly selected articles assessing all mentions within those articles, whereas this study first used machine learning to identify mentions, then randomly selected mentions for annotation. Both approaches work and shed light on our understanding of how software is cited and citable in scholarly communication.

We also compare our findings with recommendations from software citation advocates. We report these findings as a baseline for future comparison, rather than an assessment of advocacy success, because it is likely that the time frame of publications chosen is insufficient for assessing the impact of advocacy. While we chose publication dates that came after advocacy recommendations, publication timelines can be long and we do not know when these articles were drafted; some may have been drafted prior to the publication of advocacy guidelines. Second, we do not know whether the authors, editors, or venues were exposed to advocacy at all; publishing and promoting articles and principles about software citation does not mean they are widely read. Our results should be understood as relatively contemporaneous with the emergence of new software citation recommendations.

Finally, our findings about citation requests only reflect the moment of data collection and annotation. These online records are subject to constant change; this means that the citation request may have not been present when a publication that cited software was authored. Nonetheless, earlier findings were subject to this same limitation so we think the comparison between studies is useful.

### Possible explanations of changes observed

We found that mentioned software were more available and accessible, included mention of specific versions, and their source code was more frequently available, particularly as standard open source. We reason that the increase of open source and accessible source code has followed the overall rise of open source and particularly the availability of hosted platforms for software development such as GitHub. Additional pressure may also have come from funders, which increasingly signal support for open source code.

We also reason that the observed increase in the mention of software version numbers could result from both the developers’ practice and the paper authors’ practice. On the development side, more software has been produced and shared for research use. Software production in general has also increasingly followed the practices of versioning, including semantic versioning (*e.g.*, Decan & Mens, 2021) as a well established open source practice that is reinforced by code hosting platforms (*e.g.*, GitHub “releases” and git tags). On the paper author side, it seems likely that the increased prevalence of software means that authors of research papers have more awareness, and versioning may be more visible when researchers install and update software through packaging systems (including the work of resolving incompatibilities between versions or dependencies!). They may need to access online help forums such as Stack Overflow and found the version could be crucial for asking and searching for solutions. These software related practices can thus increase the saliency of versioning experienced by authors, leaving impressions of what is important to the scientific understanding of their work as well as reproducibility.

We were struck by the increase in software citation requests observed. We specifically investigated whether this overall increase was consistent across types of software, suspecting that proprietary software may make more explicit requests. However, we did not find statistically significant differences between the proportions of proprietary, non-commercial, and open source software that make citation requests, nor did we find differences with respect to how well those requests are followed by paper authors. Thus, we conclude that the practice of making citation requests is adopted by more overall. Software authors might notice prominent requests by others and then be motivated and educated to add their own. The presence of templates for language-specific features such as the citation() method in R, and the prevalence and visibility of CITATION files at the top level of code repositories may also influence software authors’ choices and behaviors. This raises hopes for software visibility; recent promising efforts include GitHub moving citation request support to the “front page” of repositories (GitHub, 2021).

### Challenges for advocacy

Our findings suggest that standards making and advocacy efforts should take existing practices into account, charting a course from current practices to hoped-for futures. For example, for researchers using software, we argue that the “like instrument” approach should be taken as a starting point for recommendations, such as re-purposing the location field for software repositories rather than a meaningless geographic location of a software publisher.

Our results about software archiving, general metadata availability, and persistent identification also have further clarified directions in which advocacy efforts can propose specific changes for different classes of software in terms of their property rights. We found over half of the open source software was archived and uniquely identified, with metadata available to fulfill citation needs. Some open source software projects were also aware of requesting citation to the software artifact directly, but largely they were still cited by their companion publications. In such cases, citing an available publication is probably a convenient choice, which is more compatible with a researcher’s conventional authoring workflow. As Bouquin et al. (2020) found that citation requests could be inconsistent when multiple online presences of software exist, recommendations can suggest concrete steps for making them actionable communications of citation expectations. The recommendations in Katz et al. (2021) & Katz et al. (2019) are a key step to ensuring that guidance begins at current practices for different kinds of software.

Another challenge rests with the citation requests of proprietary software (such as SPSS). Our observations of these requests included many that we thought almost unusable or incompatible with reference systems, including long-winded legalistic requests filled with® and ™ symbols and disclaimers of warranty; they seemed to be written by lawyers rather than specialists of scholarly communication. Advocacy in this area might therefore need to address lawyers or encourage developers to take ownership of these requests. An alternative might be for citation style guides to provide translation principles for these sorts of requests.

The persistence of non-machine readable citation requests (*e.g.*, free-text CITATION files) might also be a starting point for recommendations, such as providing migration paths and perhaps automatic synchronization between manual and machine-readable requests, meeting software producers where they are. Recent efforts are moving in this direction, including highlighting built-in language features for citation and advocating for more languages to include these (*e.g.*, Katz et al., 2021).

Maintenance costs are a likely challenge which advocacy should address. While the first step of adoption is not easy, as the efforts by Allen (2021) showed, making software and its citation human- and machine-actionable in the long term requires the upkeep of these formats and practices. One technical reason why the software artifact is recommended over its publication(s) as a citation target is that software is a very dynamic object. A single publication at one time cannot credit all those who contributed to it throughout its lifecycle. However, formal citation of the software artifact in a both machine- and human-actionable manner also raises the requirement for the upkeep of citation metadata and persistent identifier as the software artifact keeps to evolve. Bouquin et al. (2020) also found that the discoverable citation requests could become outdated when new releases of the software come out. It is likely that a non-trivial amount of regular work is needed for a software project to keep themselves citable and communicate the up-to-date citation expectations to their users.

Advocacy around persistent identifiers could also directly address the question of automated identifiers *vs.* manually created identifiers. This became clear to us as we reasoned around how to include the Software Heritage identifier in our annotation. The current archiving mechanism of Software Heritage is designed as a response to concerns of computational reproducibility (Alliez et al., 2020; Cosmo, Gruenpeter & Zacchiroli, 2020). By its endeavor to archive all software source codes (Di Cosmo, 2018), Software Heritage archives source codes crawled from major code hosting platforms. Software Heritage archives copies, with automatically generated identifiers and metadata, which can be cited as part of the research workflow reported in publications. They are open to researchers’ citation use and technically fulfill the advocacy recommendations for software citation; although they do not necessarily reflect the software creators’ preference. In contrast, the manual creation of identifiers is a costly exercise that indicates that someone thinks them worth using. Language-specific software metadata and metadata stored in third party registries are also alternative sources of citation information, and advocacy might consider how researchers can be guided to use these available resources to cite software. However, in face of multiple sources, researchers need to be able to identify the appropriate record. We found, when searching for identifiers in systems that create them automatically, it was very difficult for users to identify the canonical archived repository for a package as distinguished from archived repositories of end-user code that forked the canonical repository (or even repositories that merely used the code). These issues are parallel to the recent discussion about non-creator-instigated software identification (Katz, Bouquin & Chue Hong, 2019), commonly concerning appropriate use of third party created software identification information.

The absence of persistent identifiers for proprietary software, whether manually or automatically created, also suggests a need for advocacy to address this specifically. Current infrastructures and policies that support software archiving and identification mechanism are designed primarily for open source code (*e.g.*, Research Data Alliance/FORCE11 Software Source Code Identification Working Group et al., 2020). As with citation metadata and requests, it may be easier for third parties to create and maintain these, rather than relying on influencing commercial software publishers. Indeed, software registries have already stored public accessible metadata and even suggested citations for proprietary software. Nonetheless, proprietary software are closed source and reuse would depend on purchase and be limited. The right for third-party verification and replication of the embodied methods or any future work directly built upon them is simply not granted. Neither do their commercial publishers need academic credit. As Alliez et al. (2020) and Cosmo, Gruenpeter & Zacchiroli (2020) have well distinguished the need for citation from that for reference, citation of the overall proprietary software project may be sufficient and the aspiration of referencing the specific software artifact may be unneeded.

Finally, our experience during the data collection for this study mirrored the reality in the age of “data deluge”: While it is promising that a variety of software metadata are growing, accurate and comprehensive metadata retrieval is not straightforward for either humans or machines. Software publishers may post their citation requests across online locations using different formats and request citations of different publications for a single piece of software across its version history. This adds the challenge of identifying linkages between software and publications additional to the challenges of software identification (Hata et al., 2021).

## Conclusion

In this study, we examined a sample of software mentions automatically extracted from PDFs of a large corpus of coronavirus research published since 2016. We manually examined a stratified random sample, validating the extraction infrastructure with a false positive rate of 5%. We annotated our validated software mentions using an existing coding scheme extended with recommendations from recent advocacy, demonstrating agreement in its use among multiple annotators. In this way, we examined how software is mentioned, what functions they realize, and to what extent they conform to advocacy recommendations for software citation practices. In addition, we searched online to find and assess data about software packages, including if and how they make citation requests.

We found improvements when compared with prior studies of software mentions. We found increased mentions of software versions, increased adoption of open source software, and improved software accessibility. We also found over half of the open source software was archived, uniquely identified, and had metadata available, ready for citation needs. Finally we found a greater proportion of projects made a specific citation request.

On the other hand, other practices had not improved, or even moved in the other direction, compared to previous studies and advocates’ recommendations. We found only a few formal citations directly to the software artifact and very little use of persistent identifiers. Crediting the authors of software in the text was still rare, mostly due to informal mentions. Worse, most mentions that do credit authors were of proprietary software, which are less likely to need to receive credit in order to keep maintaining the tool. Citation requests have potential to improve this situation, but we found these to be followed only in few cases.

Organically established practices may provide appropriate starting points for advocacy. For example, existing practices for software citation differ between proprietary, non-commercial, and open source software. Our findings emphasize the long-standing practice of “like instrument” mentions primarily for proprietary software, as well as divergent practices in the identification, archiving, and metadata provision of software with different property rights. Finally, they also differ in the common locations and widely used formats for citation requests. Advocacy may be more effective by leveraging these existing practices to minimize the behavioral change needed.

Future research suggests itself in three areas: publication type, decisions about software citation, and examining change over time in relation to advocacy.

Different publication types may have different patterns of software mentions. While we stratified the sample by impact factor and did not find any significant differences, we did not separate by article type. For example, it is perhaps worthwhile to separate publication venues specializing in publishing “software papers” that describe a software application or an algorithm; their software mention and citation patterns could be different from other domain science publications. Research could also separate pre-prints, or work to identify the differences among more granular fields and sub-fields.

Future research is needed to understand decisions about software citation and their pathway to publications. Citation requests do not yet seem effective, nor do we know much about the decisions leading to this ineffectiveness. For example, requests may not be visible to authors, or not visible at the right time. Even efforts to follow requests in a manuscript may be undermined by style guides, journal instructions, reviewers, or editors. Advocacy may not align well with the pressures involved during the production and publishing of articles. Studying collections of drafts over time through the article creation process could create greater understanding of the manner in which mentions and reference lists are created and open up new locations or emphases for advocacy.^4^ 4For example, in preparation of this publication, reviewers pointed out a missing URL in a dataset entry in our reference list. Even with our heightened attention given the topic of this paper, this error made it into the PDF; inquiry showed that the initial URL entry in the BibTeX had become masked in the toolchain from BibTeX through to the PeerJ LaTeX template and citation style specified for PDF rendering. We used a work-around in BibTeX to make the URL reference visible.

Finally, future research should examine change over time, seeking evidence about effective advocacy. We hope that our extraction infrastructure, sampling approach, and content analysis scheme can be useful for comparable studies. Researchers can process future collections of PDFs produced by different groups, stratify, randomly sample mentions, and annotate and compare results to observe change. These observations could be focused on specific advocacy efforts, including micro efforts, such as comparing the publications of those exposed to specific training or interventions with those unexposed. Groups could be individuals, classes of individuals (such as software producers or early-career scholars), or larger groups such as users of specific software, classes of software or techniques, or specific fields. Assessments of interventions should be designed to give sufficient time for interventions to operate; timing of citation requests can be then compared to the period in which a study was authored and published.

Understanding patterns of software citation from research publications over time can inform advocacy and policy-making efforts to improve the visibility and rewards of software work in science. Our study suggests that software citation practices have not changed substantially in the past five years. Advocating for change takes time and we hope these results can provide a baseline against which to measure change in the future. Nonetheless, we think it possible that the behavior change required to implement new forms of visibility for software in publications may be too complex for quick uptake. Automated solutions that do not require behavioral change, such as entity extraction from PDFs to build software impact indexes, clearly have a role to play, both as a resource for improving advocacy and as a fallback for visibility where publication practices are slow to change.

## Supplemental Information

10.7717/peerj-cs.1022/supp-1 Supplemental Information 1 Full coding scheme and instructions for annotating software mentions

## Additional Information and Declarations

### Data Availability

The following information was supplied regarding data availability: The raw data is available at Zenodo: Patrice Lopez, Caifan Du, Hannah Cohoon & James Howison. (2021). Softcite software mention extraction from the CORD-19 publications (0.3.0) [Data set]. Zenodo. https://doi.org/10.5281/zenodo.5235661. The analysis code is available at GitHub: https://github.com/caifand/cord19-sw-analysis.
