# Supplementary material for: Understanding progress in software citation: a study of software citation in the CORD-19 corpus
Source: PeerJ Comput Sci. 2022 Jul 25;8:e1022. doi: 10.7717/peerj-cs.1022 (PMC9454791; doi:10.7717/peerj-cs.1022)
Supplement: Supplemental Information 1 [file peerj-cs-08-1022-s001.pdf]

Hint	Code ID	Codes	Explanation
Check the extracted mention context.	A1	The extracted `context` mentions software.	A specific computational product that needs to be instantiated by code to realize the reported research in the publication. Examples include specific computational models or algorithms that are implemented according to the context. Exceptions include a general analysis method being mentioned but is not necessarily implemented by some form of code. If the extracted context does not mention software, then code all the rest codes as "0".
	A2	The mention is a "like instrument" mention.	A "like instrument" mention of software refers to software in the manner that scientific instruments are usually being referred in papers: names of the instrument/software in text, then follow the name of the vendor and often their geographical location. e.g. "SAS 9.2, SAS Institute, Cary, North Carolina".
Check the extracted software name.	A3	The name of the mentioned software is given in context.	
	A4	The extracted `software_name` is the name of the software mentioned in context.	A3, A4 are checked together to understand if the extraction works properly to get the right information out of the context. Same below for A5-A6, A7-A8, A9-A10.
Check the extracted version.	A5	The version/release date of the mentioned software is given in context.	
	A6	The extracted `version` is the version of the software mentioned in context.	
Check the extracted publisher.	A7	The publisher/developer of the mentioned software is given in context.	
	A8	The extracted `publisher` is the publisher/creator of the software mentioned in context.	
Check the extracted URL.	A9	The URL of the mentioned software is given in context.	
	A10	The extracted `URL` is the URL of the software mentioned in context. The URL can be a resolvable identifier.	
Check the extracted mention context.	A11	Additional configuration details of the software are given in context (e.g., operating environment; language platform; parameter settings)	
	A12	The context is sufficient to know the software is used by the authors in their research.	
	A13	The context is sufficient to know the software is not used by the authors in their research.	
Check the extracted reference.	B1	If the software mention has reference extracted from bibliography in `tei`:	No coding is required for this code. If the software mention does not have an extracted reference, code B2 through to B12 as "0".
	B2	The extracted reference is the reference of the mentioned software.	This is judged by whether the reference addresses the software itself, or methods implemented in the software. Look for the referenced publication if needed. If the extracted reference is not the one presented in the original publication, then code B3 through to B12 based on the actual reference in the original publication. Otherwise, paste the actual reference in B8 unless it does not exist.
	B3	The reference is a "software publication" or a publication that discusses the software as the primary subject matter.	This might include publications that discuss software as the implementation of certain algorithm/methods. Examples also include dataset paper that substantially discusses software utilities that accompany the dataset.

	B4	The reference is the software itself in any form (e.g., source code; container; executable) or its metadata. (i.e. not a publication or any kind of document but software as a cited object)	
	B5	The reference is a domain science publication.	This might include publications that merely discuss algorithms/methods even as computational procedures, but not a concrete code product. For instance, publications in computer science or bioinformatics commonly discuss algorithms or programming methods.
	B6	The reference is a software manual/user guide.	
	B7	The reference is a project but not the software product.	
	B8	Paste the reference string from the publication if the extracted one is not correct (otherwise leave the cell empty or code as "0"):	
	B9	The extracted reference string contains the name of the software.	B9-B12 are included to understand reference as part of the software mention and whether the reference provides additional information about the software mentioned in text.
	B10	The extracted reference string contains version/release date of the software.	
	B11	The extracted reference string contains an URL of the software/project. The URL can be a resolvable identifier.	
	B12	The extracted reference string contains the publisher/creator of the mentioned software.	Usually if the reference is a "software publication" or discusses the software substantially, the authors are counted as publishers/creators of the software.
Citation functions.	C1	The software is an identifiable entity given the extracted mention.	The software is identifiable when it has a name. Available information online indicates its existence, even if it is not accessible.
	C2	The software has at least one findable official presence (e.g., source code, online manual, publication, or an online resource such as a metadata record or webpage that is dedicated to the software).	Use available information in in-text mention and references to search for the software for available online records.
	C3	The specific version of the software mentioned in the article has at least one findable official presence (e.g., a versioned release, documentation for a specific version, a web page, or an official release note).	Use available information in in-text mention and references to search for the software for available online records. If no version is mentioned in the extracted texts, then code C3 as FALSE.
	C4	Either or both in-text mention and references contains a unique, persistent identifier (e.g., DOI, ARK, Handle, PURL, NBN) that can resolve to the software itself and/or its metadata. (i.e., the software itself is registered with a persistent URI)	
	C5	Either or both in-text mention and references contains a commit hash that points to a snapshot of the software.	
Access to software.	C6	There is no access to the software.	The software may have an online presence, but there is no available information about how to access the software for use.

	C7	There is only purchase access to the software.	If the publisher offers free trials for the software, but one still needs to get a paid license for using the software, then it is still counted as purchase access.
	C8	There is free access to the software.	If the software can be accessed by personal contact or direct download/fork, but there is no information indicating a payment/license fee is required for using it, then software has free access.
	C9	The source code of the software is accessible.	
	C10	The software has a permission to modify. (i.e., free software or an open source license is available; no permission if it requires personal contact)	
	C11	The software has an open source license.	
Citation request.	D1	The extracted software mention (including reference) is referred in a way that matches the citation request of the software.	This code requires finding the citation request online if available.<br>Any public information that addresses how to cite the software from the official source of the software (i.e. not a third party) is interpreted as a citation request (i.e., it may not contain phrase like "request" but specify a way to reference the software). If a citation request is not available, code D1 through to D14 as "0".
	D2	The software has a publicly accessible citation request in plain text style.	
	D3	The software has a publicly accessible citation request in BibTex format.	
	D4	The citation request is in a working repository README. (e.g., GitHub; GitLab)	
	D5	The citation request is on project/software website/webpage.	If the citation request is on a webpage dedicated to the software mentioned, then this code is TRUE. (e.g., it could be a specific page in an online software catalog, index, or repository)
	D6	The software has a publicly accessible CITATION file.	
	D7	The software has a publicly accessible CITATION.cff.	
	D8	The software has a publicly accessible CodeMeta file.	
	D9	The software has a publicly accessible, domain-specific citation request, such as R CITATION/R DESCRIPTION file.	
	D10	The citation request asks to cite the software itself.	
	D11	The citation request asks to cite a "software publication" or a publication that discusses the software as the primary subject matter.	See the explanation for B3.
	D12	The citation request asks to cite a domain science publication.	See the explanation for B5.
	D13	The citation request asks to cite a project as a whole rather than its product.	e.g., the programming language R asks users to cite the R project.
	D14	The citation request asks to a cite a non-software product other than the ones specified above.	

	D15	OPTIONAL please specify the type of the requested citation object:	
	D16	OPTIONAL please copy and paste the link to the citation request:	
Publish software.	E1	The software has at least one version published to an archival repository (e.g., Zenodo, figshare, Software Heritage).	Conduct a within-site search on Zenodo, Figshare, or Software Heritage using the software name plus possible keywords as search term. If an archival copy of the software (no matter which version) is found, then this code is TRUE. Using the link of identified working repository for searching inside Software Heritage is particularly helpful.<br>Also examine the web search results when searching for the specific piece of software using possible search terms. If an archival copy in an institutional repository or in locations mentioned above is located in the search engine results page, then this code is also TRUE.
	E2	The software has at least one version that has a unique and persistent identifier such as a DOI, ARK, Handle, PURL, or NBN.	Web search using "<software name>"+"DOI" OR "<software name>"+"ARK" OR "<software name>"+"Handle" OR "<software name>"+"PURL" OR "<software name>"+"NBN" to locate if an archival copy of the software (no matter which version) exists.<br>Also notice if any findable archival copy of the mentioned software has a unique and persistent identifier accompanied, or the software metadata contains a unique and persistent identifier.<br>If a unique and persistent identifier of the software itself has been identified (i.e., not a publication; could refer to any version) when searching for the software online, this code is TRUE.
	E3	The metadata of the software itself is publicly accessible, including the name of the software, authors/contributors, version/release date, and/or access information (e.g., not available/online location/working repository).	If a CITATION/CITATION.cff/CodeMeta/R DESCRIPTION/R CITATION file exists and is populated with metadata items that describe the mentioned software, then this code is TRUE. The metadata could also be in a language specific form or any general software metadata form other than the citation-purpose metadata.
	E4	OPTIONAL please copy and paste the link to the software metadata:	
